# A Review of Cognitive Outcomes Across Movement Disorder Patients Undergoing Deep Brain Stimulation

**DOI:** 10.3389/fneur.2019.00419

**Published:** 2019-05-07

**Authors:** Stephanie Cernera, Michael S. Okun, Aysegul Gunduz

**Affiliations:** ^1^J. Crayton Pruitt Family Department of Biomedical Engineering, University of Florida, Gainesville, FL, United States; ^2^Department of Neurology, Fixel Institute for Neurological Diseases, University of Florida College of Medicine and McKnight Brain Institute, Gainesville, FL, United States

**Keywords:** deep brain stimulation, cognition, Parkinson's disease, essential tremor, dystonia, Tourette syndrome, cognitive domains

## Abstract

**Introduction:** Although the benefit in motor symptoms for well-selected patients with deep brain stimulation (DBS) has been established, cognitive declines associated with DBS can produce suboptimal clinical responses. Small decrements in cognition can lead to profound effects on quality of life. The growth of indications, the expansion of surgical targets, the increasing complexity of devices, and recent changes in stimulation paradigms have all collectively drawn attention to the need for re-evaluation of DBS related cognitive outcomes.

**Methods:** To address the impact of cognitive changes following DBS, we performed a literature review using PubMed. We searched for articles focused on DBS and cognition. We extracted information about the disease, target, number of patients, assessment of time points, cognitive battery, and clinical outcomes. Diseases included were dystonia, Tourette syndrome (TS), essential tremor (ET), and Parkinson's disease (PD).

**Results:** DBS was associated with mild cognitive issues even when rigorous patient selection was employed. Dystonia studies reported stable or improved cognitive scores, however one study using reliable change indices indicated decrements in sustained attention. Additionally, DBS outcomes were convoluted with changes in medication dose, alleviation of motor symptoms, and learning effects. In the largest, prospective TS study, an improvement in attentional skills was noted, whereas smaller studies reported variable declines across several cognitive domains. Although, most studies reported stable cognitive outcomes. ET studies largely demonstrated deficits in verbal fluency, which had variable responses depending on stimulation setting. Recently, studies have focused beyond the ventral intermediate nucleus, including the post-subthalamic area and zona incerta. For PD, the cognitive results were heterogeneous, although deficits in verbal fluency were consistent and related to the micro-lesion effect.

**Conclusion:** Post-DBS cognitive issues can impact both motor and quality of life outcomes. The underlying pathophysiology of cognitive changes post-DBS and the identification of pathways underpinning declines will require further investigation. Future studies should employ careful methodological designs. Patient specific analyses will be helpful to differentiate the effects of medications, DBS and the underlying disease state, including disease progression. Disease progression is often an underappreciated factor that is important to post-DBS cognitive issues.

## Introduction

Deep brain stimulation (DBS) has become an area of active scientific inquiry for the treatment of movement and other neuropsychiatric diseases (1–3). Decades of research have largely focused on optimizing the preoperative evaluation, refining neurosurgical technique, advancing target selection, and improving postoperative management (4). The efficacy of DBS depends on quality clinical outcomes along with an acceptable adverse event profile. The prospect of short or long-term complications, particularly non-motor issues (e.g., cognitive changes), can dampen efficacy and enthusiasm for continued use. Information on adverse events and selection criteria can also help to better define the populations who will most benefit. Thus, careful attention must be devoted to the investigation of cognitive issues (5).

One of the most commonly reported non-motor issues that may emerge after DBS surgery for movement disorders has been neuropsychological dysfunction, including cognitive and emotional changes. DBS outcomes can be hindered by negative neuropsychological outcomes and by mild decrements revealed in detailed testing. These deficits may have demonstrable effects on quality of life (5–7). However, cognitive decline is a complex topic and may be associated with disease progression in many movement disorders such as Parkinson's disease (PD).

To progress toward a more precise understanding of cognitive decline after DBS surgery, we conducted a detailed review of the DBS literature focusing on cognitive outcomes across movement disorder cohorts. Separate cohorts are addressed in dedicated sections with neurosurgical target in subsections. We present an overview of current evidence to elucidate the present state of the field and to motivate improved methodological design of future studies, analyses, and devices. Consequently, improved surgical techniques, novel devices, expanding indications, and complex device management issues all may be impacted by cognitive issues.

## Method

A PubMed search was conducted using the keywords “cognitive effects,” “executive function,” “cognition,” “neuropsychology,” and “neuropsychological” along with “deep brain stimulation.” The retrieved abstracts as well as their references were reviewed for relevant studies. Studies focusing on dystonia, Tourette syndrome (TS), essential tremor (ET), and PD were included. Studies which included both preoperative and postoperative cognitive outcomes were included. Studies which included only postoperative assessments or acute tests (i.e., DBS on/DBS off) were considered if the testing was performed at least 3 months after lead implantation in order to control for postoperative cognitive dysfunction. Case studies were excluded, unless applied to less common implantation sites or diseases. Table 1 summarizes the included cognitive domains and relevant tests associated with each domain.

**Table 1:** Cognitive domains and associated tests.

**Cognitive domains**	**Tests**
General cognition	Mini-Mental State Examination, Mattis Dementia Rating Scale, Reading subtest of the Wide Range Achievement Test, Montreal Cognitive Assessment, Parkinson Neuropsychometric Dementia Assessment
Attention, processing speed and working memory	Paced Auditory Serial Addition Test, Digit Span—WAIS/WMS, Letter-Number Sequencing—WAIS/WMS, Elevator Counting—Test of Everyday Attention, Self-Ordered Pointing Test, Benton Visual Retention Test, Arithmetic—WAIS, Digit Ordering Test, Conner's Continuous Performance Test, Trail Making Test—A, Symbol Digit Modalities Test, Coding—WISC/WAIS, Symbol Search—WISC/WAIS, Letter Cancellation—WISC/WAIS, Brief Test of Attention, Alertness—TAP, The “A” Test
Executive function	Wisconsin Card Sorting Test, Controlled Oral Word Association Test, Stroop Color-Word Test, Trail Making Test—B, Tower of London, Temporal Rule Induction, Frontal Assessment Battery, Delis-Kaplan Executive Function System, Go/nogo Test—TAP
Verbal memory	Hopkins Verbal Learning Test, Verbal learning—WRAML, Verbal Learning and Memory Test, Paired Associate Learning, Stories and Word Pairs—WMS/CMS, Rey Auditory Verbal Learning Test, Bi-syllabic Words Repetition Test, California Verbal Learning Test, Grober and Buschke Test
Visual and spatial memory	Faces—WMS/CMS, Dot Locations—WMS/CMS, Block Span—WMS, Nonverbal Learning Test, Recognition Memory for Faces, Corsi's Block Tapping Test, Complex Figure Test, N-back Task, Figural Memory—WMS, Brief Visual Memory Test
Language	Boston Naming Test, Graded Naming Test, Complex Ideational Material Test
Visuospatial perception	Judgement of Line Orientation, Hooper Visual Organization Test, Visual Object and Space Perception, Clock Drawing, Block Design (non-verbal)—WISC/WAIS, Copying Drawings, Line Cancellation, Multi-features Target Cancellation, Benton Facial Recognition Test, Constructional Praxis
Verbal fluency	Semantic Fluency, Letter Fluency, Phonemic Fluency, Category Fluency, Alternating Fluency
Intellectual ability	Vocabulary (verbal)—WISC/WAIS, Block Design (non-verbal)—WISC/WAIS, Matrix Reasoning (non-verbal)—WISC/WAIS, Picture Concepts (non-verbal)—WISC/WAIS, Picture Completion (non-verbal)—WISC/WAIS, Similarities (verbal)—WISC/WAIS, Comprehension (verbal)—WISC/WAIS, Multiple Choice Vocabulary Test, Graded Difficulty Arithmetic Test, National Adult Reading Test, Leistungsprüfsystem
Abstract reasoning	Raven Color Matrices, Raven Progressive Matrices
Motor speed and coordination	Halstead-Reitan Finger Oscillation, Luria's Fist Edge Palm Test, Grooved Pegboard, Sequential and Simple Tapping, Purdue Pegboard

*WAIS, Wechsler Adult Intelligence Test; WMS, Wechsler Memory Scale; CMS, Children's Memory Scale; WRAML, Wide Range Assessment of Memory and Learning; TAP, Test Battery for Assessing Attentional Disorders*.

## Dystonia

Fifteen studies (12 globus pallidus internus (GPi), 2 subthalamic nucleus (STN), 1 GPi/ventralis intermedius (VIM) nucleus of the thalamus) reported the cognitive effects of DBS for primary and secondary dystonias. Contained within these studies, 243 patients were included in the analyses. In some studies, participants were unable to complete assessments due to disabilities from the disease state, thus, the number of participants completing each task could not be reported as absolute, especially in pediatric DBS cases (8–10). We summarize the cognitive tests administered and the significant changes reported within each dystonia paper (Supplementary Table 1).

### Globus Pallidus Internus

Overall, cognitive measurements in chronic GPi stimulation remained stable among the 12 identified studies. No changes in cognitive battery were observed in Vidailhet et al.'s multi-center, prospective trial of 13 patients with dystonia-choreoathetosis cerebral palsy. This study examined measures of general intellect and executive functions one year after surgery (11). Two larger prospective trials that only used measures of global cognition [e.g., Mattis Dementia Rating Scale (DRS) and Mini-Mental State Exam (MMSE)] also reported no significant changes from pre- to post-DBS (2, 12). Improvements were observed in tests assessing memory (13, 14), cognitive set shifting (13), perceptual reasoning (8–10, 14), processing speed (15), verbal comprehension (8, 10, 14), verbal fluency (16), and executive function (14, 17).

Motivated by the limited cognitive battery used and varying results reported within previous GPi-DBS studies, de Gusmao et al. published a prospective study involving 12 patients with either primary or secondary dystonia. The authors reported a considerable improvement in their cohort post-DBS (average of 13.1 months) compared to pre-DBS on the Letter-Number Sequencing test of the Wechsler Adult Intelligence Scale (WAIS) or the Wechsler Intelligence Scale for Children (WISC) (a test of working memory). The cohort also experienced an improvement in Trail Making Test-B (TMT-B), which is a measure of executive function and processing speed, specifically cognitive set-shifting. There was a trend toward a decrease in semantic verbal fluency. There were no other evident changes on tests evaluating visual memory, language, and higher order visual processing (13). Improvements were noted in one retrospective review of 40 children with secondary dystonias who received bilateral GPi-DBS implants. The cohort had a substantial improvement in Picture Completion scores of the WAIS\WISC (9). Pillon et al. attributed post-DBS improvements in concept formation and reasoning [Raven Progressive Matrices (PM38)], executive function [Wisconsin Card Sorting Test (WCST)], and memory (Grober and Buschke Free Recall) to a reduction in anticholinergic medication. Anticholinergic therapy has been shown to be associated with a deleterious effect on memory and information processing (14). Another paper reported that individuals whose medication was unchanged after DBS experienced decrements in reaction times compared to subjects with medication reduction (15). Additionally, improvements within other cohorts were attributed to medication reduction, to the lessening of dystonic burden, and to compounding practice effects (9, 13–15). Although in one study whose only group level cognitive change was a significant improvement in Trail Making Test-A (TMT-A), the authors indicated that individual *post-hoc* analyses revealed both improvements and declines across the cognitive battery, stressing the importance of the need for both tailored therapies and reporting individual scores (15).

While some patients undergoing GPi-DBS for dystonia experienced improvement, several studies utilizing calculated methodologies [i.e., Reliable Change Indices (RCIs)] did not describe such results. RCI is a statistical measure that determines whether or not a change is clinically significant according to an individual's state before the initiation of therapy by considering a test measurement's reliability (18). In Jahanshahi et al.'s follow-up investigation of 14 patients with bilateral implants for primary generalized dystonia, the authors observed a worsening in the scaled score on Digit Span, fewer items recalled on Rey Auditory Verbal Learning Test (RAVLT), and a notable increase in errors on the Paced Auditory Serial Addition Test (PASAT). After calculating RCIs for each of these scores to determine which ones were statistically reliable, only the increase in errors on PASAT was significant. This result suggested a decrease in sustained attention in this cohort of patients, although the cohort did improve in tests of executive function, specifically on Stroop Color and the WCST (17). In another randomized, multi-center sham-controlled trial with 13 cervical dystonia patients, the only cognitive test that demonstrated detriments after 12 months was the number of words produced on alternating categories, which is a verbal fluency task. The authors hypothesized that this impairment could be due to an interruption of fronto-subcortical circuits (i.e., dorsomedial GPi), which are involved in cognitive flexibility, caused by either current spread from DBS or a micro-lesion from electrode insertion (19). Interestingly, in a follow-up analysis from Gruber et al., patients with tardive dystonia tended to improve in category verbal fluency up to 7 years after surgery (16), suggesting that a decline in verbal fluency could be a micro-lesion rather than stimulation induced effect.

Within the only paper that reported bilateral implants in both the GPi and VIM for patients with myoclonus-dystonia, no change was observed within the cognitive battery, which included tests of general cognition, reaction time, executive function, working memory, verbal memory, processing speed, and verbal fluency (20). Patients were assessed at baseline (pre-surgery), 6 months, 12 months, and long-term at an average of 62.3 months. At these follow-ups, patients were also assessed in the following stimulation patterns (VIM/GPi): OFF/OFF, OFF/ON, ON/OFF, OFF/OFF. These stimulation patterns demonstrated a substantial difference between simple reaction time, a test used to assess alertness, with impairment observed in GPi in relation to VIM stimulation. These results suggested that stimulation may have a mild effect on cognitive outcome, or on specific cortical loops influenced by either the GPi or the VIM (assuming DBS leads are optimally placed).

### Subthalamic Nucleus

Two investigations focused on cognitive outcomes in dystonia patients treated with STN-DBS. In Kleiner-Fisman et al.'s case series, four idiopathic dystonia patients experienced declines in executive function, verbal memory, visual memory, and language skills; however, no statistical testing was performed. As a whole, these patients were already impaired at baseline in multiple cognitive domains (21). In a prospective pilot study, 9 cervical dystonia patients were implanted with bilateral STN leads. Patients were impaired at baseline on tests for information processing speed (TMT-A and -B) and verbal delayed recall. Cognition was stable within 12 months after DBS implantation, suggesting that impairments in executive function and verbal fluency observed in STN PD patients may be due to underlying circuitry abnormalities inherent to PD, rather than stimulation or micro-lesion effects on the STN (22).

## Tourette Syndrome

Eight studies (3 GPi, 4 Centromedian-parafascicular (Cm-Pf), 1 GPi/Cm-Pf) reported the cognitive effects of patients undergoing DBS for TS. Within these studies, 52 patients were included in analyses. We summarize the cognitive tests administered and the significant changes reported within each TS paper in Supplementary Table 2.

### Globus Pallidus Internus

All studies assessing the effect of GPi-DBS in TS patients revealed no change in assessments from baseline to follow-up (23–25). In Smeet and colleagues' open-label study with five TS patients, tests in attention, working memory, verbal fluency, and executive function were stable between preoperative and postoperative assessments (12–38 months) (25). In one case study, no change was observed at one year in the cognitive tests Verbal Learning Memory Test and Stroop, which are measures of verbal memory and executive function, respectively (23). Finally, Kefalopoulou et al.'s double-blind, randomized crossover trial in 15 bilateral patients demonstrated no alterations in cognitive functioning between baseline and open-label conditions; however, there was a significant effect of time on the California Verbal Learning Test (CVLT) Immediate Recall, on which patients performed worse in off-stimulation conditions (24).

### Centromedian-Parafascicular Complex

Ackermans et al. explored the cognitive effects of DBS in a case study of two patients with follow-ups of 6 and 10 years. Case 1 (10-year follow-up) had stable scores in measures of verbal and non-verbal memory, executive function, mental speed, and attention. Case 2 had variable outcomes over the course of 6 years. This patient experienced post-operative worsening in letter verbal fluency, total numbers learned in 5 trials of the RAVLT, and a substantial increase in the time to perform the Stroop task, which eventually returned to baseline at 6 years (26). Although only two cases, this paper demonstrates the differential outcomes that can be observed under similar DBS paradigms, suggesting both the practicality of personalized stimulation paradigms or devices and the potential advantages of reporting individual outcomes rather than group averages. Ackermans et al. continued exploring this topic in a double-blind, randomized controlled trial, where there was a significant increase in the time required to perform the Stroop Color Word Test (SCWT) one year after DBS, which suggested a decrease in response inhibition and selective attention. The authors proceeded to perform RCIs, which concluded that only one patient performed worse in the SCWT (27). Much like Jahanshahi's analyses, RCIs explained which factors or patients drove significance and *post-hoc* tests proved essential to better appreciate the true effects of DBS (17).

To further eliminate confounding factors such as learning effects, Schoenberg et al. conducted a prospective, randomized trial with 4 TS patients, where they utilized alternate test forms. At baseline, the cohort was impaired in TMT-B, the written version of Symbol Digit Modalities Test, Continuous Performance Test (CPT-2) hit rate, and SCWT. At 5 months, the group demonstrated impairments in these measures as well as RAVLT-total words, letter fluency, and semantic fluency. The authors conducted Cohen's *d* tests to observe the effect sizes of these deficits. Deteriorations in semantic and phonemic verbal fluency were large, whereas the declines in CPT-2 hit rate and immediate memory from the visual memory task were moderate. Additionally, the improvement observed on the visuo-constructional skill task (Complex Figure Test) was a medium sized effect (28). Another prospective study found no changes in 15 patients after 24 months with bilateral implants in the Cm-Pf ventralis oralis anterior area except for an improvement on TMT scores. However, this paper did not explore measures of sustained attention or verbal memory (29). The differences in findings from these two studies suggested a potential micro-lesion effect from DBS surgery, which was demonstrated in the immediate deficits captured from Schoenberg's investigations. Furthermore, the opposing results could have been attributed to the heterogeneity found between the two studies in the neuropsychological battery, implant area, sample size, or statistics. For instance, Porta's analyses used Wilcoxon-Signed Rank Tests; whereas, Schoenberg used standardized paired *t*-tests, corrected for multiple comparisons, and controlled the small sample size using false discovery rate.

Finally, Welter et al.'s double-blind, randomized, controlled, crossover trial reported the cognitive results of 3 TS patients with bilateral implants in both the GPi and the Cm-Pf. Neuropsychological battery remained stable between preoperative and postoperative follow-ups. The follow-ups were 2 months after surgery without stimulation, followed by four different stimulation conditions, which were applied and sustained for 2 months. The stimulation conditions were bilateral Cm-Pf, bilateral GPi, both bilateral GPi and Cm-Pf, and sham. Although this experiment involved stable cognitive functioning, conclusions should be approached with caution due to low sample size (30).

## Essential Tremor

Six studies [1 caudal zona incerta (cZi), 2 ventrolateral nucleus (VL) of the thalamus, 3 VIM] reported the cognitive outcomes of patients following DBS for ET. Additionally, one analysis compared VIM-DBS in ET patients with STN-DBS in PD patients, whereas another study compared stimulation of the VIM between ET, PD, and multiple sclerosis (MS) cohorts. The complete cognitive batteries administered, and results have been summarized in Supplementary Table 3.

### Caudal Zona Incerta

Fytagoridis et al.'s prospective pilot trial investigated the effects of DBS on verbal fluency in 17 patients at baseline and off stimulation at 3 days. There were also 10 patients tested at one year both on and off stimulation. There was a considerable reduction in verbal fluency 3 days after surgery, but this effect dissipated at one year both on and off stimulation. Therefore, this may have been a micro-lesion effect, however the sample size was too small to determine (31).

### Ventrolateral Nucleus of the Thalamus

In their open-prospective study, Heber et al. conducted a series of neuropsychological tests on 9 ET patients implanted into the VL region of the thalamus. The subtest “Alertness” of the Test for Attentional Performance was used to assess patients. This subtest is a simple reaction time test that requires a patient to press a button upon detecting a visual stimulus. The patient performs four blocks, in which two blocks consist of no warning tone before the visual stimulus appears and two blocks consist of a warning tone before the stimulus appears. At one year, the patients were remarkably slower with DBS-OFF compared to both pre-surgery and DBS-ON, specifically in the blocks without warning tone. Using *post-hoc* analyses, the authors demonstrated that the differences between DBS-ON and -OFF were statistically different, whereas differences between DBS-ON and -OFF against pre-surgery reaction times were negligible. These results were consistent at 6 years as well. Tests of verbal fluency, memory, executive function, and intellect were preserved at 1 and 6 years after surgery. The authors noted that the surgical electrode trajectory did not impact reaction time tests, and those patients who had implantations through supplementary motor area and through other cortical entry points did not differ (32). Another investigation evaluated the acute effects of stimulation settings (i.e., high frequency vs. low frequency) on measures of verbal fluency (parallel versions), executive function, and working memory. There was a difference in both measures of verbal fluency under different stimulation conditions. Low frequency stimulation led to both better phonemic and semantic verbal fluency compared to high frequency stimulation (33). Similar results were demonstrated in a group of STN-DBS PD patients, where 10 Hz stimulation hindered motor improvement but improved verbal fluency (34). Since low frequency stimulation exacerbated tremor and high frequency suppressed tremor, Pedrosa et al. concluded these results potentially supported the idea of segregated networks for motor control and for higher cognition (33).

### Ventralis Intermedius Nucleus of the Thalamus

In Tröster et al.'s outcomes study (*n* = 40), which compared baseline scores to 3 month post-operative scores, there were significant improvements in DRS-Construction subtest, visual span backwards, Hooper Visual Organization Test, Grooved Pegboard, Delayed Word Recognition of the CVLT and Delayed Prose Recall, measured by Logical Memory II of Weschler Memory Scale (WMS) (35). The only significant decrement was observed in lexical verbal fluency, however, concurrently, there was an improvement on the communication score measured by the Parkinson's Disease Questionnaire-39, which is a quality of life scale. Although most of the group level comparisons demonstrated improvement in scores, individual analyses revealed reductions on the DRS subscales Attention, Initiation, and Perseveration. Additionally, the authors speculated that improvements in visual attention, working memory, and visuoperceptual functioning may have been caused by thalamic stimulation facilitating an attentional gating mechanism, therefore, stimulation aided in filtering out extraneous information and enhanced interhemispheric information transfer. This hypothesis could additionally support Heber et al.'s finding of improved reaction time during on stimulation trials compared to off (32). In a tandem study, Fields et al. investigated the cognitive outcomes at 12 months in mostly the same cohort as Tröster's outcomes study (36). All improvements were maintained at 12 months, with additional improvements in CVLT Immediate Recall, Short-Delay Recall, Long-Delay Recall, and Recognition Hits from baseline to 12 months, and in CVLT Immediate Recall and DRS Conceptualization scores from 3 to 12 months. Although, the authors stated that the gains observed may be due to practice efforts. In terms of cognitive declines, lexical verbal fluency remained diminished at 12 months, with 4 additional patients demonstrating declines in semantic verbal fluency.

Determined to tease apart the underpinnings of cognitive decline, one study separated patients who experienced cognitive decline after DBS from those who did not (37). The authors defined those who had decrements (ET-D) as patients who decreased by one standard deviation compared to baseline assessments in one or more cognitive tests and in at least two domains of function, which included global cognitive functioning, attention, executive function, language, visuoperception, and learning and memory. This study demonstrated that ET-D patients did not have more severe tremor and were not significantly older or cognitively lower functioning at baseline. ET-D patients had significantly higher pulse width settings and were more likely to have undergone left hemisphere DBS compared to stable participants. Patients with greater pulse width settings (> 120 μs) were 10 times more likely to exhibit postoperative cognitive decline, which the authors attributed to current spread into adjacent VIM association fiber tracks. Additionally, pulse width settings and age at disease onset accurately predicted whether a patient was in the stable or decrementing cognitive group. These results demonstrate the attention to detail that must be utilized within the clinic to safely and effectively determine programming settings. Furthermore, these results highlight the importance of patient selection to ultimately minimize the risk of cognitive deficits.

### Comparative Studies

One paper investigated the differential effects of stimulation on verbal fluency in patients with PD (STN), ET (VIM), and healthy controls (38). Both DBS groups uttered fewer words when compared to healthy controls, however there were no substantial differences between the DBS cohorts. There was a considerable effect of task demand (i.e., phonemic vs. semantic). When comparing DBS-ON vs. -OFF, there was a significant interaction between group and stimulation state. *Post-hoc* analysis revealed that there was a notable reduction in the number of words produced during DBS near the VIM, particularly in phonemic fluency. Conversely, DBS in the STN improved phonemic fluency. The error rate, specifically the types of “wrong category” and “word stem repetition,” was also substantially reduced by VIM stimulation. Furthermore, Ehlen et al. investigated the correlations of these outcomes in STN stimulation. Stimulation amplitude and the electrode trajectory were key predictors for the change in phonemic fluency, in which higher stimulation amplitude and more anterior locations correlated with better verbal fluency. The authors speculated that stimulation within the STN restored impaired left fronto-cortical functions. These same predictor variables were included in the VIM, but increasing stimulation caused decreased verbal fluency. Another relationship uncovered was that electrodes located more posterior and dorsolateral were associated with better verbal fluency scores, thus, electrode trajectories may have influence on cognitive outcomes (38). Similarly, Loher et al. investigated the effects of stimulation within the VIM in PD, ET, and MS patients. Stimulation deteriorated the number of words recalled on the short delay recall of the RAVLT in all groups, and demonstrated an alteration in episodic memory, which was related to left-sided stimulation and altered simple reaction times (39). These results verified that in this subset of patients, episodic memory was influenced by stimulation and not a micro-lesion effect. Additionally, impairments in frontal lobe tests (Stroop, verbal fluency, Go/nogo of the Test Battery for Attentional Disorders), constructional praxis, and cognitive processing speed (Alertness of the Test Battery for Attentional Disorders) were observed under stimulation off and on conditions, and changes were most evident in the PD cohort. These studies ultimately stress the importance of truly delineating the underlying causes of cognitive declines post-DBS.

## Parkinson's Disease

There are numerous papers investigating the cognitive side effects following DBS for PD, and we have divided the summary into the following sections: outcome studies with a control group, outcome studies without a control group, correlation studies, studies that included new DBS techniques, and studies that compared the outcomes of GPi- vs. STN-DBS.

Within the literature search, 19 studies (all STN) included a control group, 29 studies did not include a control group (24 STN, 4 GPi, 1 VIM), 10 (9 STN, 1 GPi) were correlation studies, 3 included either new stimulation or surgical techniques for DBS (1 STN, 2 GPi and STN), and 12 compared STN and GPi outcomes. Within the controlled studies, 650 DBS patients were included with 433 controls (40 with DBS implants). Within studies without a control group, 704 (60 GPi, 9 VIM) DBS patients were included. Correlation studies included 304 (14 GPi) patients, new technique studies included 160 patients (25 GPi) with 65 controls, and studies that compared pallidal vs. subthalamic outcomes had 519 GPi patients and 579 STN patients. Information regarding the cognitive assessments utilized and the outcomes are in Supplementary Tables 4–8, respectively.

### PD Outcome Studies With a Control Group

In studies that followed both patients that had undergone DBS and patients solely being treated with drug therapy, DBS patients either experienced declines in performance over time that were not evident in controls or were significantly impaired when directly compared to controls, namely in the following cognitive domains: verbal fluency (40–49, 51–55), executive function (40, 45–49, 51–54, 56), general cognition (49, 51, 54, 55), visuospatial reasoning and memory (49, 53), processing speed (53, 56), and verbal memory (45–47, 49, 51, 56). In a two-year follow-up analysis, STN-DBS patients exhibited impairments on tasks involving non-verbal recall, processing speed, and verbal fluency (both phonemic and semantic). A trend was observed for problems with SCWT. The authors used RCI to draw conclusions solely based on the effects of DBS on cognition and to delineate these effects from PD progression. After computing RCIs, the percentages of patients in both the STN-DBS (*n* = 19) and PD control group (*n* = 18) that deteriorated on non-verbal recall, processing speed, phonemic verbal fluency, semantic verbal fluency, and executive function were 47 vs. 25%, 53 vs. 28%, 26 vs. 11%, 29 vs. 29%, and 43 vs. 18%, respectively (53). Within the 6-month outcomes, the STN-DBS group deteriorated on verbal delayed recall and verbal fluency when compared to PD controls. When the authors considered age of onset, education level, and dopamine dosage, the worsening of verbal fluency was negligible, even though 26% of patients in the STN group performed worse on the task compared to only 4% of the controls (56).

In a similar long-term analysis, Tramontana et al. noted that DBS patients had deficits in phonemic fluency and on several subtests of the WCST at two-year follow-up compared to controls. However, when the authors eliminated patients who suffered from an adverse event in the DBS cohort, these differences were trivial (52). Sáez-Zea et al.'s prospective, controlled study found a correlation between more reduction in medication and a greater reduction in phonemic verbal fluency (48). Similarly, Smeding et al. reported that decreases in DRS and the Auditory Verbal Learning Test were correlated to low levodopa at baseline, emphasizing the importance of preoperative screening for optimal patient outcomes (51). Additionally, the STN group performed worse on all measures of verbal fluency, on Attention and Initiation of the DRS, on delayed recall, and on SCWT compared to controls at 6 months, although, apart from delayed recall (verbal memory), these declines were not due to negative side effects from surgery, electrode misplacement, or reduction in medications. Thus, the authors stated the outcomes may be linked to executive dysfunction stemming from PD. All these papers collectively indicate the importance of controlling for confounding factors when analyzing the cognitive effects of DBS, and the importance of patient selection.

There were some instances when the DBS group either outperformed or remained stable in comparison to the control group. In one analysis, controls tended to perform slightly worse in TMT-B at follow-up. In addition, the authors found a correlation between higher age and an increase in time to complete TMT-B, which they attributed to PD progression (48). In Zangaglia et al.'s long-term controlled study, the authors observed trends for improvement on Verbal Span, Digit Span, Corsi's Block Tapping Test, and Logical Memory Test, which are all measures of memory at 3 years after surgery; whereas, controls had a considerable decrease in WCST and MMSE at 3 years. Although there was a trend toward increased scores in memory assessments, the authors stated that it could have been a learning effect that masked deterioration since alternate versions were not used. Furthermore, the test results were confounded by impairments noted in the WCST (55). Finally, when using RCIs, Williams et al. observed a significant interaction for clock drawing, a visuospatial task. PD controls tended to become more impaired at 2 years with 47% declining in contrast to only 16% in the STN-DBS group (53). However, in one investigation, visuospatial functioning was impaired in both groups at one year (45), and notably impaired only in the STN-DBS group at one year in another analysis (49). These results support the notion that treatment needs to be tailored toward the patient, and that more emphasis needs to be placed on follow-up times, neuropsychological batteries used (i.e., alternate tests), and how to control for confounding factors.

Although most studies reported in this review had control groups that were PD patients on optimal medical therapy, a few studies focused on other comparisons. For example, two studies focused on the underlying cognitive differences after DBS and pallidotomy (57, 58). In Gironell and colleagues' 6-month outcomes study, STN-DBS patients declined in semantic verbal fluency, whereas they remained stable in measures of executive function (SCWT and TMT-B) (57). However, in another study, STN-DBS patients at 6 months experienced an increase in the total number of errors on SCWT and TMT-B, while the control group demonstrated improvement (58). Additionally, the increase in errors on SCWT was significantly correlated with lower baseline DRS scores at 6 months post-operatively, further demonstrating that cognitive changes can be heavily influenced by the individual patient and test battery. Finally, Merola et al.'s retrospective observational study classified one group as normal cognition STN-DBS patients (*n* = 134) and another as mild-cognitively impaired (MCI) STN-DBS patients (*n* = 40). Both patient groups were comparable at their follow-up times in tasks quantifying visuospatial functioning, memory, and processing speed, except for one-year follow-up, where normal cognition patients performed worse on phonemic verbal fluency. The authors credited this result to the baseline of the MCI group which revealed significant impairment. Though the two groups were comparable on neurocognitive assessments, the MCI group had a markedly lower estimated time until dementia (6.03 years) compared to 11.08 years in the normal cognition group (50). These results support that STN-DBS is cognitively safe, even when used to treat patients that are mildly impaired.

### PD Outcome Studies Without a Control Group

When analyzing studies not including a control group, impairments observed were remarkably similar to DBS patients within controlled studies. DBS patients exhibited deteriorations after surgery compared to preoperative performances in tasks of verbal fluency (59–77), memory (59, 62, 64, 66–68, 71, 72, 75, 77), executive function (59, 60, 64, 67, 69, 71, 72, 76–80), attention (66, 71), visuospatial functioning (59, 72, 75), global cognition (62, 74, 78), abstract reasoning (62), and processing speed (64, 72, 76, 77). A few studies observed no cognitive changes up to 3 months (81), in which individuals who did decline were significantly older, had higher levels of levodopa at baseline, and all had left implants in the GPi, up to 6 months (82, 83), and up to 5 years (84), in which there was a trend for a decline in verbal fluency. Within other studies, the outcomes of verbal fluency were variable. Some authors described an improvement, albeit not to baseline levels, of verbal fluency in the long-term compared to an initial substantial reduction in scores, supporting the possibility of a micro-lesion effect (60, 70, 76). In Lefaucheur et al.'s short-term outcomes, patients had an acute significant reduction in verbal fluency 3 and 10 days post-operatively, however their scores had a reliable improvement at 6-month follow-up (70). In another study, patients had a significant reduction at one month on both semantic and phonemic verbal fluency, but phonemic completely recovered and semantic was improved at the 12-month follow-up (76). However, most studies reported verbal fluency impairments one or more years later after DBS as compared to baseline (61–63, 65–67, 69, 71, 72, 77, 85), suggesting disease progression rather than lesion effects. In GPi-DBS outcomes papers, most studies did not identify a reduction in verbal fluency, with the exception of one (74), suggesting the possibility that the STN and related circuits may have a more substantial role in verbal fluency processing (78, 81, 86).

In four studies that followed patients post-operatively 5 years or more, patients had a significant decrease at 5 years on total and Perseverative Errors on the WCST (executive function) (77), verbal fluency (62, 77, 85), Raven's color matrices (reasoning) (62, 77), and delayed recall of the RAVLT (memory) (85). In Kishore and colleagues' study (*n* = 47), there were no significant cognitive declines at 5 years, however, when analyzing individual scores, there were 10 patients who declined in verbal fluency compared to one at baseline (84). Similarly, individual analyses revealed several cognitive declines that were not observed in Contarino et al.'s group assessments (62). At their long-term follow-ups, 8 (85) and 9 (77) years, patients had deteriorations in the Bi-syllabic Words Repetition Test (BWR) (77), TMT-B (77), verbal fluency (77, 85), and Immediate Recall on the RAVLT (85). In Zibetti et al.'s study, dementia developed in one patient at one-year, 2 patients at 5 years, and 4 patients at 9 years or more (77). These decrements could possibly have been due to disease progression.

Many studies reported deficits in executive function and memory. In Rizzone et al.'s 12-year long-term follow-up, patients had a significant worsening in contrast to baseline on short-term memory (Corsi's Block Test Forward), episodic memory (Immediate and Delayed Recall on the RAVLT), executive function (WCST) and attention (Attentive Matrices). The authors attributed these findings to be expected in advanced PD patients, especially since 22.7% of patients developed dementia in their cohort (71). Another investigation with a one-year follow-up initially reported a notable impairment on tasks of executive function (Stroop) but the scores eventually recovered, although were considerably worse than baseline measures (76). Heo et al.'s one-year follow-up study also reported a substantial reduction on both tasks for verbal memory and Stroop test at both 6 and 12 months (67). These effects were not solely in STN-DBS patients with Bonenfant and colleagues reporting a significant worsening in SCWT and Stroop Interference at 3 years in comparison to baseline within a GPi-DBS cohort. Although the authors reported stable scores on the WCST, there was an overall reduction in general cognition (78). One study observed an improvement in memory, which the authors attributed to practice efforts (73) and another investigation observed increased memory until one year after the surgery followed by deficits at 5 and 10 years (69). Similarly, one study reported improvement in TMT-B in 24 unilateral STN patients (87). Interestingly, in the only study involving the VIM in PD, there were significant improvements in Delayed Recognition of the CVLT and Delayed Recall of WMS-Logical Memory (88), although the authors stated that they could not demonstrate if these improvements were clinical relevant. The heterogeneity of these results reveal the complexity of PD post-DBS. Such variations in outcomes within and across studies may relate to age, disease duration, medication, neuropsychological instruments, electrode localization, and time of follow-up and reassessment. These factors should be controlled and considered, especially in studies lacking a control group.

### Correlation Studies

Many studies investigated the influence of the following factors on neuropsychological outcomes: volume of tissue activation (VTA), white matter lesions (WML), electrode trajectory, active contacts, brain perfusion, and microelectrode (MER) tracks. One retrospective study explored the relationship between deficits in verbal fluency and number of MER passes, and concluded that there were no correlations between PD duration, MER passes, baseline cognition, stimulation parameters and verbal fluency. However, verbal fluency scores were correlated with age (89). Mikos et al. investigated the relationship between VTA, which represents neuronal activation, within the STN and verbal fluency (alternate forms) in 17 PD patients (90). The stimulation paradigms examined were no stimulation, optimal stimulation, ventral stimulation, and dorsal stimulation. There were no differences in verbal fluency scores among the three electrode contacts, but other relationships were reported. Optimal stimulation correlated positively with VTA inside the STN and letter fluency change scores, meaning more VTA within the STN was associated with better fluency scores compared to off stimulation, which corroborated results from Ehlen and colleagues' study (38). However, with ventral stimulation, there was a negative association with VTA and STN, implying that a larger volume of VTA inside the STN was associated with worse letter fluency performance relative to off stimulation. These relationships were not observed with category fluency, which the authors attributed to category fluency relying more on the temporal lobe. Whereas, letter fluency relies more on fronto-subcortical structures with an abundance of projections to the STN, making letter fluency potentially more susceptible to stimulation (90). This assertion was the opposite of what Cilia and colleagues reported using brain perfusion imaging, where they noted that decrements in category fluency were related to hypoperfusions in dorsolateral prefrontal and anterior cingulate, both frontal lobe regions, in addition to the ventral part of the caudate and premotor cortex (91). However, Mikos' study demonstrates that a reduction in verbal fluency may not only be due to surgical impact, but also influenced by stimulation. Interestingly, these methods were repeated in 14 GPi patients, and no significant relationship was discovered between the magnitude or location of VTA and verbal fluency performance (92). This finding supported the possibility that GPi stimulation and surgery impact verbal fluency less than STN. Bonenfant et al.'s study was supportive of this idea (78).

In Blume et al.'s retrospective review focusing on WML, 40 patients with bilateral STN implants were analyzed. The authors developed a cognitive composite score (CSS) to correlate cognitive dysfunction with WML. All tests scores were transformed into *z*-scores by averaging the scores of five domains (attention, executive function, language, memory, visual-constructive). After 3 years in 17 patients, substantial reductions were reported in semantic verbal fluency, TMT-A, and the Block Design Test. Fifteen of these patients fulfilled the criteria for PD-MCI or PD dementia (PD-D), in which 10 patients developed PD-D 3 years after DBS with four occurring within the first post-operative year. The only considerable differences between PD-D and non-demented patients were age and occurrence of hallucinations at baseline. WML were associated with age and one or more cardiovascular risk factors. Patients who developed PD-D had a higher volume of WML at baseline compared to non-demented patients. Likewise, a worsening of CSS was correlated to the volume of WML after correction for age in a linear regression analysis (93). This study demonstrated that declines in cognition could be influenced by several factors.

Five studies investigated STN electrode trajectory or contacts. One study considered if lead trajectory involving the caudate was correlated with cognitive dysfunction. TMT-B decreased substantially more in the caudate involved group in contrast to the group that did not have caudate disruption at 3 months. At 12 months, TMT-B was markedly reduced in both groups with a greater decrease in the caudate involved group. Verbal fluency notably worsened in both cohorts compared to baseline assessment. Since performance was decreased in both groups, these results contradict the hypothesis that caudate involvement has a substantial effect on verbal fluency (94). In Witt and colleagues' lead trajectory analysis, patients who exhibited decrements on DRS and Digit Span Backwards had trajectories that were more medially located which resulted in a greater overlap in the caudate nucleus compared to stable performers. Whereas, stable performers had more lateral trajectories, resulting in greater lesions within the basal ganglia, specifically the globus pallidus. Patients that worsened on both Stroop task and semantic verbal fluency had electrode positions outside the stimulation area of the left STN, which, for semantic verbal fluency, confirmed the results of Mikos et al.'s investigation that more VTA within the STN region resulted in a better performance on verbal fluency (90, 95). Additionally, patients who performed worse in semantic fluency had ventral electrodes positioned in the left STN. This result was similar to Smeding et al.'s case study, where ventral contact activation in both hemispheres demonstrated declines in verbal fluency, but this effect was lessened after dorsal contact stimulation (96). Ventral stimulation in the STN has been speculated to produce more cognitive and mood-related effects, since the sensorimotor region is located posterior and dorsolateral (97). However, the authors noted that the ventral contacts were located outside the STN, namely placed within the internal capsule and dorsomedial globus pallidus externus (96).

York et al. found that if a patient's ventricles, not the caudate nucleus, were involved within the DBS lead trajectory, they demonstrated greater impairments on verbal long-term memory and verbal fluency following DBS surgery. Declines in MMSE, DRS, long-term verbal memory, short-term verbal memory, verbal fluency and semantic fluency were correlated with electrodes placed more lateral in either hemisphere, superior in the left, posterior lateral in the left, lateral in the right, posterior and superior in the left hemisphere, and superior in the right, respectively (98). One study found that patients who had trajectories with a more anterior cortical entry, which ultimately spared or passed through less of the thalamus, had greater reductions on semantic fluency, while there were no relationships between lead trajectory and phonemic verbal fluency (99). Finally, Floden et al. explored the relationship between active contact and cognitive alterations. Semantic fluency decreased with more medially located active contacts in the left hemisphere; whereas, phonemic fluency decreased with more posterior left-sided contacts. In the right hemisphere, there was a significant relationship between increasing stimulation voltage and worse single trial learning on the RAVLT (verbal memory) (100). These studies demonstrate that cognitive outcomes may be tricky to interpret and that pre- vs. post-operative scores may not be enough. In the future, directional DBS leads may be shown to be advantageous for avoiding cognitive deficits (100).

### Different Study Designs and Techniques in PD

One trial explored the effects of constant current DBS devices vs. the standard constant voltage (101) with neuropsychological outcomes reported in a second study (102). In this randomized controlled trial, 101 patients were treated with active stimulation, while 35 underwent delayed stimulation until the 3-month follow-up. At 3 months, both groups had significant impairments in category and switching fluency. The stimulation group had notable reductions on all parts of the Stroop and on letter verbal fluency, with improvements on several measures of memory; whereas, the control group had considerably worsened in the Initiation score of the DRS. At 12 months, the Vocabulary subtest of WAIS, verbal fluency and Stroop significantly declined, while measures of working memory increased (102). These results are comparable with devices using constant voltage. This study revealed that verbal fluency was primarily a surgical and not stimulation induced effect, though stimulation may also possibly be a minor factor in the decline.

Two studies explored the outcomes of using image-guided DBS instead of the traditional MER technique. In Brodsky et al.'s study, patients who underwent image-guided DBS (7 STN and 23 GPi) had a substantial improvement in category fluency at 6 months, while patients who underwent standard DBS surgery (MER-guided) had a decline in category fluency (18 STN and 21 GPi). Additionally, the difference in verbal fluency was significant between both groups. Phonemic fluency was unchanged in the asleep group but was considerably worsened in the awake group. DRS remained stable in both groups at 6 months (103). However, the sample size was too small to definitively conclude the superiority of one approach. Although, another study assessing asleep guided DBS (16 STN and 4 GPi) found a mild decrease in scores for category fluency, Complex Figure Copy and memory at one-year follow-up (104). Though, this study did not use statistical techniques. The difference between these two studies could have possibly been the time between follow-ups, selection of patients or differences within targeting methods.

### Comparison of GPi vs. STN Stimulation in PD

Whether GPi or STN stimulation offers equal motor benefits while avoiding long-term cognitive or mood side effects has been an important question within the DBS field (105). Several longitudinal studies have sought to answer this question by comparing both DBS groups (106–114), while others compared each stimulation group against one another and a control group (115–118). Determined to enhance the evidence supporting the difference in cognitive outcomes between unilateral STN (*n* = 22) and GPi (*n* = 23), Okun et al. conducted a prospective, randomized trial (111). To evaluate regional settings, the investigators stimulated under four different paradigms: ventral, dorsal, optimal, and off. In the optimal setting, the STN group had a worsening in letter verbal fluency compared to GPi, but this finding did not reach pre-defined significance. This phenomenon persisted regardless of stimulation setting, suggesting that this was an insertion or lesion effect. When observing post-surgical cognitive adverse events across groups, the GPi-DBS group had 12 (2 serious) adverse events with difficulty in speech and language, while STN had 8. Additionally, GPi-DBS had 3 adverse events in worsening of memory, whereas STN had 2, suggesting the importance of both individual and group level analyses.

In Odekerken et al.'s one-year follow-up study, bilateral STN (*n* = 56) and GPi (*n* = 58) groups notably differed on SCWT, TMT-B, and were borderline different on WAIS Similarities, which were all worse in the STN-DBS group. These results suggested STN-DBS may have a considerable effect on mental speed, attention, and language. Seventeen patients in the GPi group exhibited cognitive decline, whereas 22 patients exhibited worsening in the STN group. Moreover, the authors reported independent predictors of cognitive decline, which included age and semantic fluency at baseline (110). Within the 3-year follow-up of the same cohort, no clinically relevant differences were evident on cognitive measures between the two groups. Dementia incidence was similar between both groups, with 4 patients in the GPi group and 5 in the STN (107). In another 2-year follow-up study, the only difference between the GPi (*n* = 152) and STN (*n* = 147) groups was within the processing speed index driven by the digit symbol visuomotor task, which declined more in the STN group (108). After 3 years in the same cohorts, the groups differed substantially on the DRS between 36 months and baseline and on the Hopkins Verbal Learning Test (HVLT) total and Delayed Recall (36 months vs. 6 months and baseline), which showed no change in the GPi group (114). The authors did not adjust for differences found at baseline between the groups or other covariates. Overall, these studies demonstrated potential differences in cognition between targets.

Other studies investigated the differences between the two surgical targets and a control group. In Rothlind et al.'s prospective, randomized, controlled trial, two between group differences were observed at 6 months between GPi and STN. STN worsened to a greater extent in Stroop Word Reading; whereas, the GPi group declined more in performance on the HVLT. Since the differences were minimal, the two DBS groups were pooled and contrasted with the best medical therapy cohort. This resulted in the DBS group demonstrating greater deficits in multiple measures of processing speed and working memory. After performing RCIs, the two DBS groups considerably differed on Digit Symbol Coding, a measurement of processing speed, with 11.1% of the STN group indicating impairment compared to only 1.3% in the GPi-DBS cohort (116). The next two studies attempted to address two methodological issues within the literature, namely, lack of PD control groups and focusing solely on group mean differences. The first study focused on a specific collection of cognitive tasks that activated the dorsolateral prefrontal cortex (DLPFC), stemming from the hypothesis that current spread to the associative basal ganglia-thalamocortical loop of the GPi and STN would affect the DLPFC. The control and DBS group markedly differed on letter fluency and semantic fluency compared to baseline, but letter fluency issues persisted and were notably impaired in the DBS group even after controlling for disease duration and Unified Parkinson's Disease Rating Scale-III off-score in the analysis of covariance. In the GPi group, medication dosage change negatively correlated with change in letter fluency. Additionally, the side of surgery was significantly related to the change in semantic fluency. Patients who underwent right-sided surgery presented with an increase in performance, albeit slight, of 0.88 points; however, patients who underwent left-sided surgery experienced a decrease of 14 points. Using RCIs, only one out of 8 patients worsened on semantic fluency for right-sided surgery; whereas, 8 out of 14 patients with left-sided surgery declined on the same measure (118). In another study, there was a main effect of time for the visuospatial multivariate analysis of covariance, implying all participants (DBS and PD controls) demonstrated lower scores on visuospatial tests. *Post-hoc* analyses revealed a worsening only on the Judgement of Line Orientation, not facial recognition test. At 12 months, DBS patients performed remarkably worse on tests of processing speed. For TMT-A, there was a significant interaction between group and time, but for Stroop Word Reading, there was only an effect of time, suggesting both groups were impaired. Using RCIs, a greater proportion of DBS patients demonstrated a reliable decline from baseline to 12 months on the HVLT Immediate and Delayed Recall, TMT-A, Stroop Word Test, TMT-B, and SCWT. However, a greater proportion of DBS patients also displayed reliable improvement from baseline to 12 months on SCWT and Judgment of Line Orientation (115).

In one study, the control group was composed of patients who underwent unilateral pallidotomy (117). Across groups (left pallidotomy, STN-, GPi-DBS), there was a significant decrease in phonemic verbal fluency. Within left unilateral pallidotomy patients, a worsening of working memory, measured with Digit Span Backwards, was reported, whereas only a trend was observed in STN-DBS patients. Additionally, left pallidotomy patients were impaired on verbal learning, specifically total score of the CVLT. Pallidotomy patients also improved in attention measured with PASAT. DBS, specifically STN, declined on executive functions (TMT-B), Long Delay Free and Cued Recall of CVLT, and visuospatial reasoning measured by the Battery for Memory Efficiency. The authors noted that there was a significant effect of age in the STN-DBS group, warning that patients >69 years of age are at more of a risk for cognitive changes. Overall, the authors stressed the importance of baseline cognitive status, test sensitivity, and using alternate versions. These findings emphasize the importance of controlling for these confounding effects across any type of cognitive study.

To further delineate effects of stimulation vs. surgery, Pillon et al. assessed STN-DBS and GPi-DBS patients while the stimulators were both on and off 3 to 12 months post-DBS (112). Improvements in Graphic Motor Series, SCWT, TMT-A, and TMT-B were noted in the STN-DBS cohort, whereas no differences were marked in DBS-on and -off states for GPi. The authors attributed the improvements in the SCWT, TMT-A and -B to improvements in psychomotor speed, since no significant changes were noted in cognitive speed for Stroop Interference or the difference between TMT-A and -B. In a similar study by Jahanshahi et al., PD patients were assessed on several tests of executive function off-stimulation, on-stimulation, and then off-stimulation, again (109). While stimulation was off, there were no significant differences between bilateral STN- and GPi-DBS groups. While stimulation was on, the authors found four different outcomes within their neuropsychological testing results. Both STN and GPi stimulation demonstrated improvements in TMT-A, TMT-B, their difference, Paced Visual Serial Addition Test, missing digit, and Control of SCWT compared to off-stimulation conditions. For conditional associative learning, both STN- and GPi-DBS deteriorated performance. STN and GPi also demonstrated different outcomes on TMT-B, TMT difference, Perseverative Errors of the WCST, and measures of random number generation, which in all cases, STN substantially improved responses. The authors speculated that this result stems from STN's differential impact on DLPFC, compared to GPi. Finally, stimulation did not change results on verbal fluency and on measures of seriation within random number generation. The authors did caution that chronic DBS may have different cognitive outcomes compared to this study, since subjects were assessed 2–26 months after surgery (109). These studies were successful at measuring the acute effects of DBS with fairly similar results for STN- and GPi-DBS outcomes, but chronic studies have shown decrements rather than improvements in the same or similar neuropsychological tests within the STN (108, 110, 115, 117).

## Conclusion

**Figure 1:**
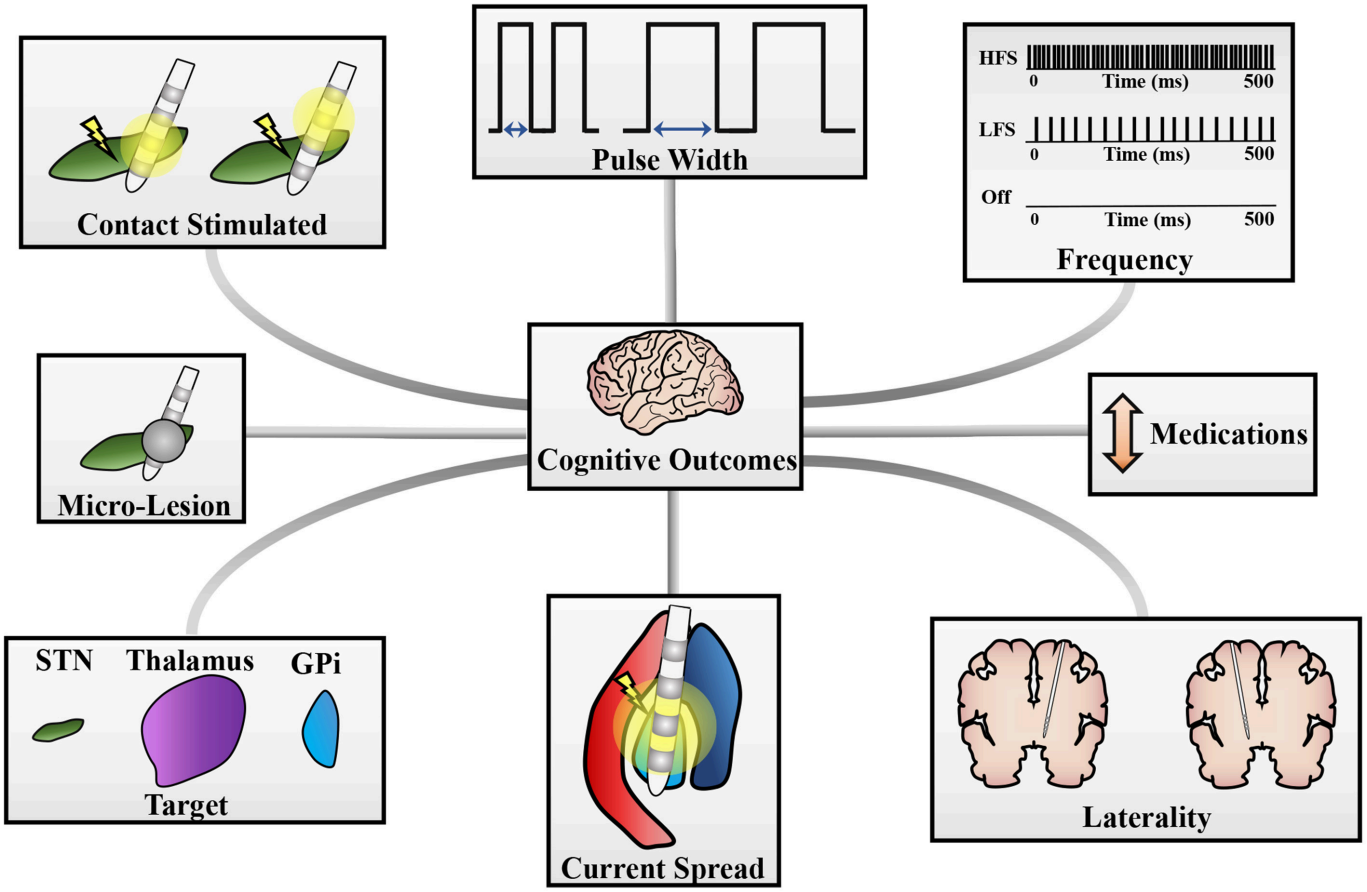
Potential sources of cognitive changes post-DBS. Cognitive changes can stem from a variety of sources and pictured here are a few potential contributors to these changes. These include (clockwise): pulse width of stimulation, frequency of stimulation, changes in medication doses, laterality of DBS implantation, current spread to neighboring structures (i.e., GPi stimulation spreading to the internal capsule), target stimulated for therapy, a micro-lesion effect, or the contacts stimulated on the DBS lead [adapted with permission from figures originally published in Eisinger et al. (125)].

DBS therapy has mixed cognitive outcomes across studies, targets, and methodologies. The expansions to new indications such as Alzheimer's disease (119) or addiction (120), to various age groups (121, 122), and to novel surgical targets (123, 124) should prompt a consideration of the factors that may lead to cognitive decline. Overall, this review highlights the lack of large, well-controlled and powered studies reporting cognitive effects of DBS and highlights heterogeneity in methods. Additionally, it emphasizes the various contributions to cognitive alterations (Figure 1). The pathophysiological mechanisms of cognitive modifications post-DBS are intricate and individually variable, consequently, the evidence provided in this review can only partially delineate the true factors involved in cognitive ramifications. The primary DBS targets for movement disorders are within the basal ganglia, a set of nuclei linked to cortical areas (i.e., DLPFC, lateral orbitofrontal, and anterior cingulate) through several cortico-striato-thalamo-cortical loops (126, 127), which are known to not be anatomically separate; thus, these disorders present with a myriad of symptoms, including cognitive dysfunctions (128, 129). Additionally, DBS may propagate through these loops, initiating modifications of influenced brain circuits, increasing the difficulty in pinpointing the true causes of cognitive dysfunction post-DBS (130, 131).

From several electrophysiology studies, it has been speculated that dystonia arises from increased inhibition of both the STN and GPi by inputs from the globus pallidus externus, causing disinhibition of the thalamus and increased excitation to the cortex (132). Pathophysiology of cognitive modifications in dystonia after DBS is not concrete, but a few theories have been postulated to explain potential dysfunctions: anti-dystonic medications affecting memory (133, 134), concurrent mood disorders (i.e., depression or anxiety) leading to impairments in executive function or other cognitive domains (135–138), or severe motor impairments shadowing intact cognitive functioning (139, 140). Altogether, evidence suggests that dystonia patients have intact global cognition, language and memory, while isolated incidents of impaired executive function and sustained attention may stem from fronto-striatal abnormalities (137, 140, 141). The DBS studies reviewed do not recall potential cognitive circuits disrupted during DBS, but many conclude that changes post-DBS are congruent with a decrease in anti-cholinergic medication (14, 15), a lessening of burden from suppressing motor symptoms of dystonia (9, 13), already present executive dysfunction or impaired sustained attention (17), or practice effects of the task (9, 13). The evidence in this review for dystonia fails to separate effects of DBS and of the aforementioned factors. However, studies attributed decreases in verbal fluency to unspecific stimulation spread to neighboring structures, especially the dorsomedial GPi, disrupting the fronto-subcortical circuit, or to a micro-lesion effect (13, 16, 19). Altogether, the evidence suggests that DBS improves other burdens of dystonia (i.e., medication dose, motor fluctuation severity), which in turn improves or worsens cognition within dystonia cohorts. Although, this review does not separate the cognitive outcomes based on dystonia type, thus, the conclusions may not be accurate across the variations of dystonia. Dystonia studies could have benefited from a control group, which would be necessary to correct for confounding factors such as disease progression, aging, re-test efforts, and even teasing out stimulation or lesional effects.

Similar to dystonia, TS is thought to arise from disinhibition of thalamo-cortical circuitry due to decreased activity of the striatum causing excessive activation of fronto-cortical areas (142). Overall, the cognitive profile of TS has been associated with deficits of executive function, inhibitory control, and cognitive flexibility; however, these aspects are convoluted with comorbidities such as attention deficit disorder and obsessive-compulsive disorder, which can exacerbate neurocognitive impairments. Thus, it is difficult to disentangle the causes of such impairments within TS cohorts (143, 144). The DBS studies within this review reported stable cognitive scores after GPi-DBS (23–25) and some impairments reported after thalamic DBS, yet, many of these impairments were driven by one patient (26, 27) or convoluted by baseline cognitive impairments (28). Studies also reported stable cognitive functioning after thalamic DBS (29) and after both thalamic and GPi implants (30). Therefore, DBS seems to have a minimal effect on cognition in TS cohorts, but this can be due to bias from the studies sampled. To make more conclusive findings about DBS and TS, neuropsychological papers reporting DBS outcomes should attempt to separate groups based off comorbidities or severity of tics, since both of these factors can influence cognition (143, 145). Another limitation is the lack of control groups within TS studies.

ET was once thought to be a monosymptomatic condition, but reports have emerged describing cognitive deficits including problems with verbal fluency, memory, mental set-shifting, and executive function (146–150). These deficits have stemmed from various pathophysiological mechanisms including abnormalities in DLPFC through the thalamo-cerebellar loop (147), an underlying clinical cerebellar syndrome (151), or pathological oscillations disturbing the normal physiological dynamics of the nervous system (152). ET-DBS has been thought to exhibit little to no cognitive impairment in chronic studies, but the studies within this review reported minor reductions in verbal fluency (32, 33, 35, 36, 38), which could ultimately stem from already abnormal cerebello-thalamo-cortical loops underlying verbal fluency or stimulation spreading to cerebellar pathways (37). Interestingly, Pedrosa et al. reported that this phenomenon is frequency dependent, and could not simply be a micro-lesion effect since phonemic and semantic fluency were differentially modulated (33). Furthermore, Heber et al. reported no impairments in verbal fluency, although the authors stated that they used lower stimulation amplitudes compared to previous studies (32), suggesting that current spread was limited. These conflicting results welcome techniques, such as patient-specific VTAs, that could potentially be useful for understanding the underlying thalamo-cortical circuitry or fiber tracts affected by DBS (90, 92, 153). Additionally, the minimal decrements observed in ET-DBS within in this review may be accounted for by the location of the sensorimotor regions within the thalamus (lateral) compared to both the limbic and associative territories (97). There has been a paucity of studies focused on ET-DBS and cognition, and only one study within this review utilized a control group (38). With recent trials now examining targets beyond VIM including the posterior subthalamic area (154) and Zi (155), ET studies should expand their methodologies and correlations to consider influences such as changes in medication dosage, disease duration, and age to adequately assess the benefits and risks of each target. These considerations will be critical for future clinical trials.

Cognitive decrements in PD are heterogeneous in several regards, including the severity of impairment and the cognitive domain affected. These deficits have been well-reported, reviewed, and are comprised of reductions in memory, executive function, attention, language, and visuospatial functioning, resulting from degeneration of nigro-striatal dopaminergic neurons and subcortical abnormalities, ultimately interfering with frontal lobe functions through under activation (156, 157). Similarly, these cognitive issues are associated and heavily researched within DBS cohorts (50). Interestingly, the cognitive results were heterogeneous across the various studies, which is already observed in PD patients without DBS. However, declines in verbal fluency were observed in most studies similar to ET-DBS. Verbal fluency was clearly a surgical implantation effect, with patients demonstrating an initial reduction in scores that returned to near baseline levels (60, 70, 76), though variation in stimulation parameters and location could also worsen outcomes (34, 90). There was a substantial difference in cognitive outcomes between STN- and GPi-DBS studies in regard to the amount of declines post-DBS, and this difference could have manifested from several factors. The STN and GPi are both basal ganglia nuclei with separate anatomical sensorimotor, associative and limbic areas, but the STN is sufficiently smaller compared to the GPi. Additionally, the aforementioned anatomical regions comprise about one-third of the nuclei within the STN, whereas the sensorimotor region within the GPi spans 53% of the structure (97). Therefore, unspecific current spread is easier to evoke in STN-DBS, potentially influencing cognitive circuits traversing in the nuclei's associative region (72). Furthermore, studies have primarily focused on STN-DBS compared to GPi-DBS, which could be another factor contributing to STN being associated with more frequent cognitive declines. However, in studies that directly compared the two targets, STN had a greater frequency of cognitive declines (107, 108, 110, 114). To add to the complexity of this debate, Ostrem et al. reported no cognitive dysfunction after STN implantation in dystonia patients, attributing PD-DBS decrements in executive function and verbal fluency to underlying circuit malfunctions (22). While, Merola et al. concluded that STN-DBS is safe for even MCI PD patients, supporting the idea that other factors are being overlooked in the search for understanding and quantifying cognitive dysfunction (50). Although there have been numerous studies attempting to quantify the cognitive effects of PD-DBS, important factors still need to be revised and further considered including follow-up times, surgical techniques, postoperative management, cognitive battery, and statistical methodologies. More investigations should be completed and should focus on relationships between cognitive outcomes and correlations such as VTA, electrode trajectory, and activated DBS contacts, since these investigations will be invaluable when mapping the networks affected. Furthermore, there has been emerging evidence of PD patients presenting with different cognitive subtypes, thus, separating different DBS patients into their appropriate subtypes may provide substantial meaning to group average cognitive comparisons (158–162).

Stemming from the lack of studies and various contributions, there is an urge to design larger, well-controlled, and sufficiently powered clinical studies to describe the effects of DBS on cognition, to refine and potentially standardize appropriate candidates for DBS, and to define criteria that substantiates or reflects what true clinical cognitive change is (163, 164). Additionally, there has not been a unified agreement of when exactly motor improvement is acceptable at the expense of cognitive dysfunction. The current standard of analyzing cognitive outcomes in DBS cases is still subpar especially if we want to reliably understand and report cognitive issues in post-DBS cohorts. Subsequently, cognitive issues can limit stimulation effectiveness, thus limiting the therapeutic window of DBS and negatively impacting quality of life. Although cognitive DBS issues and data have been available for more than a decade, the underlying pathophysiology of cognitive declines post-DBS will need further investigation. The identification of relevant pathways could lead to better device design and implementation (e.g., directional leads). This review stresses the importance of patient specific analyses and accurate lead localization, since there can be differential outcomes of DBS in similar cohorts (i.e., importance of defining patient criteria). Moreover, this review raises the question as to whether the results on a group level represent clinical significance, since even minor changes in cognition can advance a patient into a state of severe dysfunction (6). However, the data presented here are only descriptive findings and a formal meta-analysis may lead to a more precise understanding between cognitive declines and DBS. Finally, we should reflect on how we can better track cognitive changes in daily situations rather than using only a single test. Implementing these changes may help us to better understand true cognitive DBS related alterations.

## Author Contributions

SC conceptualized the paper and wrote the first draft, developed the first draft of figures and tables, and finalized the paper, figures and tables. SC, MO, and AG provided inputs and edits.

### Conflict of Interest Statement

The authors declare that the research was conducted in the absence of any commercial or financial relationships that could be a potential conflict of interest.
