# Supplementary material for: A Review of Cognitive Outcomes Across Movement Disorder Patients Undergoing Deep Brain Stimulation
Source: Front Neurol. 2019 May 7;10:419. doi: 10.3389/fneur.2019.00419 (PMC6514131; doi:10.3389/fneur.2019.00419)
Supplement: Supplementary file 1 [file Table_1.DOCX]

Within these tables, only significant outcomes are described unless otherwise stated.

| **Supplementary Table 1. Dystonia Cognitive Outcomes** | | | | | | |
| --- | --- | --- | --- | --- | --- | --- |
| **Author** | **Target** | **N** | **Type of Study** | **Evaluation Time** | **Tests Performed** | **Outcomes** |
| de Gusmao et al.,  2017 | GPi | 12 | Outcomes study | Baseline and 12 months | DS (WAIS/WISC), Letter Number Sequencing (WAIS/WISC), TMT-A, SDMT, COWAT, TMT-B, SCWT, BNT, SVF, HVOT, Verbal Learning (WRML), Faces (WMS/CMS) | 1) Improvements in WAIS-IV letter-number sequencing (*p* = 0.024)  2) Improvements in TMT-B (*p* = 0.032)  3) Trends towards decrease in SVF  - Significance calculated using one-tailed student *t*-test, *p*-value ≤ 0.05 significant |
| Owen et al.,  2017 | GPi | 40 | Retrospective longitudinal study | Baseline and 1 to 3 years | Block Design, Matrix Reasoning, Picture Concepts, Picture Completion, Vocabulary, Similarities, Comprehension, Faces and stories, DS, and Letter Number Sequencing | 1) Increase in WASI Picture Completion (Z = -1.97)  - Significance calculated using Wilcoxon sign-ranked test, *p*-value ≤ 0.05 significant |
| Dinkelbach et al.,  2015 | GPi | 13 bilateral | Randomized, sham-controlled, multicenter trial | Baseline and 12 months | Verbal Learning and Memory Test, DSF, Block Span – Forward (WMS), Nonverbal Learning Test, DOT, LVF, SVF, Elevator Counting (TEA), Stroop Task, JLOT, Visual Object and Space Perception (Object Perception, Number Location, Cube Analysis), Graded Difficulty Arithmetic test, Multiple Choice Vocabulary Test | 1) Decrease in numbers of words produced in alternating SVF (*p* = 0.020)  -Significance calculated using Wilcoxon signed-rank test, *p*-value ≤ 0.05 significant |
| Owen et al.,  2015 | GPi | 13 bilateral | Retrospective study | Baseline and 1-3 years | WISC-IV (Perceptual Reasoning, verbal comprehension), WASI (Block Design, Matrix Reasoning, Picture concepts, Picture Completion, Vocabulary, Similarities, Comprehension), Faces Dot Locations, Stories, and Word Pairs (CMS/WMS), DS and Letter Number Sequencing, Coding, Symbol Search, Cancellation | (Stable, Improved, Declined)  1) Verbal Comprehension Index (4,4,1)  2) Perceptual Reasoning Index (7,3,1)  3) Working Memory Index (6,2,0)  4) Processing Speed Index (4,2,1)  5) Visual immediate memory index (6,1,0)  6) Visual delayed memory index (4,1,1)  7) Verbal immediate memory index (3,1,1)  8) Verbal delayed memory index (4,1,1)  -Powered statistics not used |
| Jahanshahi et al.,  2014 | GPi | 14 bilateral | Follow-up study | Baseline and 1 year or more | MMSE, NART, WAIS (verbal IQ), RAVLT, Recognition Memory for Faces, Self-ordered Pointing Test, PVF, SVF, GNT, MWCST, SCWT, TMT, PASAT | 1) Decrease in WAIS-R Digit Span (*p* = 0.024)  2) Decrease in RAVLT – Correct Recall (*p* = 0.022)  3) Improvement in MWCST non-perseverative errors (*p* = 0.013)  4) Improvement in Stroop control task (*p* = 0.027)  5) Increase of errors on the PASAT (*p* = 0.021)  - After calculating RCI, PASAT was the only clinically significant deterioration  - Significance calculated using *t*-test, *p*-value ≤ 0.05 significant |
| Gruber et al.,  2009 | GPi | 9 bilateral | Follow-up study | Baseline, 1 week, 3-6 months, long-term | MDRS, NART, Simple and pre-cued visual reaction tasks, PVF, CVF, RAVLT, DS | 1) Trend for improvement on CVF at long-term follow-up (~ 7 years) (*p* = 0.06)  - Significance calculated using Student *t*-test, *p*-value ≤ 0.05 significant |
| Vidailhet et al.,  2009 | GPi | 13 bilateral | Multi-center, prospective pilot study | Baseline and 1 year | MMSE, Raven PM-38, Similarities and Arithmetic Subsets of WAIS-R, Free and Cued Selective Reminding Test, WCST | 1) No significant effects  - Significance calculated using Wilcoxon signed-rank test for matched pairs, *p*-value ≤ 0.05 significant |
| Gruber et al.,  2010 | GPi/Vim | 10 (8 GPi/Vim, 1 GPi, 1 Vim) | Follow-up study | Baseline, 6, 12, and long-term (623 ± 39 months) | MDRS, Simple and Pre-cued Visual Reaction Task, RAVLT (parallel versions), DS, Stroop task, TMT, LVF, SVF | 1) Impaired simple reaction time in GPi versus Vim-DBS (*p* = 0.04)  - Significance calculated using Wilcoxon signed-rank test for matched pairs, *p*-value ≤ 0.05 significant |
| Mahoney et al.,  2010 | GPi | 7 bilateral | Case series | Baseline and 1-4 years | Matrix Reasoning, Picture Concepts, Picture Completion (WISC), Vocabulary, Similarities, Information and Picture Vocab, Memory for faces, dot locations, Digit Span | Participants 1-4  1) Improvement on scores of non-verbal intellectual abilities  Participants 5-6  1) Participant 5 improved on matrix reasoning, faces memory, and faces delayed recall  2) Both could not complete assessments pre-DBS due to disability  Participant 7  1) Deterioration of picture vocabulary  -DBS did not work, which the author attributed to lead location  -No statistical measure |
| Vidailhet et al.,  2005 | GPi | 22 bilateral | Prospective, multicenter study | Baseline and 12 months | MMSE | 1) No significant change  - Significance calculated using paired Wilcoxon signed-rank, *p*-value < 0.05 significant |
| Kupsch et al.,  2006 | GPi | 40  bilateral | Randomized, prospective trial | Baseline and 6 months | MDRS | 1) No significant change  - Significance calculated using paired Wilcoxon signed-rank, *p*-value < 0.05 significant |
| Halbig et al., 2005 | GPi | 15 bilateral | Case series | Baseline and 3-12 months | MDRS, Tonic and Phase Alertness, Stroop, PVF, CVF, TMT-A, TMT-B, RAVLT, DS | 1) Slight improvement on TMT-A  - Descriptive analysis revealed that individual patients showed improvement or decline  - Significance calculated using paired t-tests, *p*-value < 0.05 significant |
| Pillon et al., 2006 | GPi | 22 bilateral | Prospective, controlled, multicenter study | Baseline and 12 months | MMSE, Raven PM-38, WAIS (Similarities, Arithmetic), Grober and Buschke Test (parallel forms), WCST, LVF, CVF, TMT-A, TMT-B, TMT-B-A | 1) Improvement on Raven PM-38 (*p* = 0.04)  2) Improvement on WAIS Similarities (*p* = 0.02)  3) Improvement on free recall of Grober and Buschke (*p* = 0.03)  4) Improvement in maintaining (*p* = 0.03) and total errors (*p* = 0.02) on WCST  - Significance calculated using Wilcoxon matched pairs signed-rank, *p*-value < 0.05 significant |
| Ostrem et al., 2011 | STN | 9 bilateral | Prospective pilot trial | Baseline, 6 and 12 months | MMSE, TMT-A, HVLT (Trial 1, Total Learning, Delayed Recall), BNT, JLOT, WAIS-III (Digit Span, Letter-Number Sequencing), SVF, LVF, TMT-B | 1) No significant changes between baseline and follow-up  - Significance calculated using Friedman test followed by post-hoc Wilcoxon signed-rank test for matched pairs, *p*-value < 0.05 significant |
| Kleiner-Fisman et al.,  2007 | STN | 4  bilateral | Case series | Baseline, 3, and 12 months | WAIS-III (Digit Span, Letter-Number Sequencing, Arithmetic, Similarities, Symbol Search, Digit Symbol), WCST, SCWT, TMT-A, TMT-B, LVF, CVF (animal naming), BNT, Boston Diagnostic Aphasia Examination, Complex Ideational Material, HVLT, BVMT, Finger-Tapping Test, CDT | 1) Mild, insignificant decline in basic executive functions (n = 4)  2) Worsening of verbal memory (n = 2)  3) Worsening of visual memory (n = 2)  4) Decline of language skills (n = 3)  - Decline\improvement based on z-score and percentage change analyses |

| **Supplementary Table 2. Tourette Syndrome Cognitive Outcomes** | | | | | | |
| --- | --- | --- | --- | --- | --- | --- |
| **Author** | **Target** | **N** | **Type of Study** | **Evaluation Time** | **Tests Performed** | **Outcomes** |
| Smeets et al.,  2016 | GPi | 5 bilateral | Open-label study | Baseline and 12 to 38 months | Digit symbol coding (WAIS), digit span (WAIS), TMT, SCWT, CVF, LVF | 1) No significant changes between baseline and follow-up  - Significance calculated using Wilcoxon signed-rank test, *p*-value < 0.05 significant |
| Kefalopoulou et al., 2015 | GPi | 15 bilateral | Double blind randomized, crossover trial | Baseline, 6, 7, 19, and 31 weeks (change in scores versus on and off weeks) | CVLT, Recognition Memory for Faces, VF, TMT-B, Stroop interference test, Letter cancellation test, PASAT, CBT | 1) Significant effect of time point on CVLT, where patients performed worse in off-stimulation conditions (*p* = 0.005)  2) No other differences between on stimulation versus off stimulation  - Significance calculated using repeated measures ANOVA test, *p*-value < 0.05 significant |
| Schoenberg et al., 2015 | Cm-Pf | 5 bilateral | Pilot, prospective, randomized clinical trial | Baseline and 5 months | WRAT (Reading Subtest), CPT, Complex Figure Test (parallel forms), SDMT, RAVLT, COWAT (phonemic, parallel forms), SVF, BNT, Halstead-Reitan Finger Oscillation, TMT, SCWT, WCST | 1) Impaired on TMT-B, written task on SDMT, CPT-2 hit rate, SCWT, average score on RAVLT, LVF, SVF after 5 months of stimulation  - Trend for declines in psychomotor speed (CPT-2 hit rate) (raw *p* = 0.05, adjusted FDR-*q* = 0.70) and SVF (raw *p* = 0.08, adjusted FDR-*q* = 0.70)  - Declines in SVF and PVF were large (*d* > 0.8), CPT-2 (*d* = 0.7), and immediate memory score were moderate (*d* = 0.8)  - medium-sized effect of improved performance was found for visuo-constructional skill task (*d* = 0.51)  - Significance calculated using repeated measures ANOVA, corrected with FDR, *p*-value < 0.05 significant. Change size effect using Cohen’s *d* |
| Ackermans et al.,  2011 | Cm-Pf | 6 | Double blind, randomized, cross-over trial | Baseline and 1 year (ON STIM) | Similarities, digit symbol coding, digit span, and matrix reasoning of the WAIS, WCST, SCWT, TMT, CVF, LVF, Tower of London | 1) Increased time needed to complete the color-word card of the Stroop (*p* = 0.046)  -reliable change only showed a decline in one patient, who also showed a decrease in mental speed  - suggests a decrease in selective attention and response inhibition  - Significance calculated using Wilcoxon sign-rank, *p*-value < 0.05 significant |
| Ackermans et al.,  2010 | Cm-Pf | 2 | Case series | Baseline and 6 and 10 years | Case 1  Verbal and nonverbal memory, executive function, mental speed, and attention  Case 2  Verbal fluency (LVF), RAVLT, Reproduction scores, Stroop | Case 1  1) No cognitive declines  Case 2  1) Decline in verbal fluency (letter) and total number of words learned in 5 trials of the RAVLT  2) No change in free recall and recognition  3) Significant increase in time needed to perform the Stroop, but decreased back to baseline at 6 years  - No statistics performed |
| Welter et al.,  2008 | Cm-Pf/GPi | 3 | Controlled, double-blind, randomized trial | Baseline, 2 months without stim and then every 2 months with different stimulation settings | Verbal learning, DOT, TMT-A/B | 1) No change in cognitive performance during any condition  - No statistics performed |
| Porta et al.,  2009 | Cm-Pf/Voa | 15 Bilateral | Prospective cohort study | Baseline and 24 months | MMSE, PM47, PVF, SVF, Rey-Osterrieth Complex Figure, TMT-A/B | 1) Improved attentional skills as per TMT-A (*p* = 0.045) and TMT-B scores (*p* = 0.001)  - Significance calculated using Wilcoxon signed rank test, *p*-value < 0.05 significant |
| Dehning et al.,  2008 | GPi | 1 bilateral | Case study | NA | VLMT and Stroop | 1) No cognitive changes  - No statistics performed |

| **Supplementary Table 3. Essential Tremor Cognitive Outcomes** | | | | | | | |
| --- | --- | --- | --- | --- | --- | --- | --- |
| **Author** | **Target** | **N** | **Type of Study** | **Evaluation Time** | **Control Group** | **Tests Performed** | **Outcomes** |
| Fytagoridis et al.,  2013 | cZi | 17 | Prospective pilot study | Baseline, 3 days (-/OFF), and 1 year (-/OFF, -/ON) | NA | Verbal fluency | 1) Significant decrease after 3 days (*p* = 0.036), but not 1 year  (*p* = 0.2678)  - Statistics performed using paired *t*-tests, *p*-value < 0.05 significant |
| Heber et al.,  2013 | VL of the thalamus | 9 | Open-prospective study | Baseline, 1 and 6 years with DBS-ON and OFF | NA | Vocabulary and reasoning, Regensburger Wortflüssigkeitstest – verbal fluency, Color-Word Interference, WMS-Digit Span, WAIS-Digit Span Backwards, CBT, VLMT, Test for Attentional Performance (simple reaction test) | 1-year  1) Without warning tones, significant slower reaction times (*p* = 0.04) (compared with DBS-ON and DBS-OFF – *p* = 0.01, *d =* 0.5)  6-year  1) Without warning tones, significant slower reaction times (*p* = 0.03) (compared DBS-ON and DBS-OFF – *p* = 0.008, *d* = 0.77)  - Statistics performed using Friedman test for multiple comparisons, post-hoc test was Wilcoxon signed rank with Bonferroni adjustment, *p*-value < 0.05 significant |
| Pedrosa et al.,  2014 | VL of the thalamus | 14 bilateral | Acute study | ~4.6 years, High and low frequency stimulation, DBS-OFF | NA | Digit Span, SCWT, PVF, SVF | 1) Significant difference in performance on phonemic (*p* = 0.012) and semantic verbal fluency (*p* < 0.045) across three different stimulation conditions  - Low frequency stimulation led to better phonemic verbal fluency (*p* < 0.05) and semantic verbal fluency (*p* < 0.05) compared to high frequency  - Statistics performed using ANOVA, post-hoc test was Tukey-HSD, *p*-value < 0.05 significant |
| Ehlen et al.,  2014 | Vim | 13 | Acute study | NA | 14 PD-STN; 12 HC | Verbal fluency | 1) Main effect of group on verbal fluency (*p* = 0.008)  - both groups (VIM OFF: *p* = 0.041; STN OFF: *p* = 0.006) uttered less words than HC, but no difference between groups  (*p* = 0.370)  2) Significant interaction between semantic/phonemic and alternating/non-alternating demonstrating performance varied with task demands  (*p* < 0.001)  - significant decline in mean number of words produced induced by DBS in Vim (*p* = 0.004)  - Statistics performed using ANOVA repeated measures,  *p*-value < 0.05 significant |
| Fields et al.,  2003 | Vim | 40 unilateral | Outcomes study | Baseline, 3 and 12 months | NA | MDRS, WMS, Brief test of attention, SCWT, WCST, CVF, LVF, BNT, Grooved Pegboard, Benton facial recognition, HVOT, CVLT | 1) Five DRS subtests combined changed significantly over time (*p* < 0.001)  - Improvements from baseline to 12 months in Construction scores (*p* < 0.001) and from 3 to 12 months in Conceptualization scores (*p* = 0.05)  2) Improvement in Grooved pegboard (dominant hand) was maintained from baseline to 12 months (*p* < 0.01)  3) Improvement in HVOT from baseline to 12 months (*p* < 0.01)  4) Significant improvement on CVLT Immediate Recall, Short-Delay Recall, Long-Delay Recall and Recognition Hits (*p* < 0.01)  - Also an improvement in Immediate Recall from 3 to 12 months (*p* = 0.01)  5) Not significant, but 4 patients showed declines in SVF, and LVF was diminished  - Statistics performed using the F value associated with Wilks’ λ, α = 0.05 |
| Woods et al.,  2003 | Vim | 49 | Correlation study | Baseline and 3 months | NA | NART-estimated verbal IQ, DRS, brief test of attention, SCWT, WMS (Digits Backward, figural memory), COWAT, WCST (preservative errors, categories), CVLT, HVOT, Benton facial recognition test | -Groups were split into post-surgical neuropsychological decrements (n = 27) and neuropsychologically stable (n = 22)  Decrement vs. Stable  1) Significant decline on general cognitive functioning (*p* < 0.05), attention (*p* < 0.01), executive function (*p* < 0.01), visuoperception (*p* < 0.001), learning and memory (*p* < 0.001)  2) The ET-decrement group had significantly higher pulse widths than the ET-stable (*p* = 0.01, Cohen’s *d* = 0.81) and a greater proportion underwent left stimulation (*p* = 0.002)  2) Pulse width and age at disease onset were significant predictors of group membership (*p* = 0.003)  - Chi-square tests and MANOVAs were used to evaluate differences between stable and decrement groups,  *p*-value < 0.05 significant |
| Loher et al.,  2003 | Vim | 5 PD, 2 ET, 2 MS unilateral | Acute study | Stim ON vs. OFF at least 6 months post-op | NA | Stroop test, VF, Go/NoGo Test of the TAP, Constructional Praxis, RAVLT, Recall, Alertness of TAP | STIM OFF vs. ON  1) Subjects recall more words on short delay recall of RAVLT stim OFF vs. ON (*p* = 0.04)  - related to left side stimulation (*p* = 0.05) and altered simple reaction times (*p* = 0.04) - Significance tested using Wilcoxon tests, *p*-value < 0.05 significant |
| Tröster et al.,  1999 | Vim | 40 unilateral | Outcomes study | Baseline and 3 months post-op | NA | DRS, HVOT, Grooved pegboard, CVLT, WMS, Brief test of attention, SCWT, WCST, Cognitive estimation test, LVF, CVF, BNT, Facial recognition | 1) Significant improvements DRS-Construction subtest (*p* < 0.001), visual span backward (*p* < 0.01), HVOT (*p* < 0.01), Grooved Pegboard (*p* < 0.001), delayed word recognition of CVLT (*p* < 0.05), and delayed prose recall (Logical Memory II) of WMS (*p* < 0.01)  2) Only significant decrement was seen in LVF (*p* < 0.001)  - Significance tested using paired *t*- tests, *p*-value < 0.05 significant |

| **Supplementary Table 4. Parkinson’s Disease with Control Groups Cognitive Outcomes** | | | | | | | |
| --- | --- | --- | --- | --- | --- | --- | --- |
| **Author** | **Target** | **N** | **Type of Study** | **Evaluation Time** | **Control Group** | **Tests Performed** | **Outcomes** |
| Demeter et al.,  2017 | STN | 10 bilateral | Prospective study | Baseline (ON/-) and 4-6 months (ON/ON) | 10 PD patients awaiting DBS | MMSE, Digit Span, CBT, N-Back (2- and 3-back), Stroop test, TMT-B, PVF, SVF | 1) Significant difference between both groups on SVF  (*p* = 0.03)  - Statistics performed within subject using Wilcoxon signed-rank and between groups using Mann-Whitney U Tests, *p*-value < 0.05 significant |
| Tramontana et al., 2015 | STN | 15 | Randomized, clinical trial | Baseline, 12, and 24 months (OFF/OFF) | 15 optimal drug therapy patients | Purdue Pegboard, JLOT, BNT, PVF, SVF, Digit Span (WAIS), PASAT, word list learning and memory for faces (WMS), WCST, SCWT | 12 months between both groups  1) ODT performed better on PVF (*p* = 0.047) and SCWT  (*p* = 0.035)  2) DBS declined in PVF  (*p* = 0.036) and SCWT  (*p* = 0.002) compared to baseline  -Significant mean change from baseline to 12 months on the Digit Span (WAIS) (*p* = 0.004), WCST Perseverative errors  (*p* = 0.053), and slowest (*p* = 0.009) and fastest (*p* = 0.005) paced rates of PASAT  24 months  1) ODT performed better than DBS on PVF (*p* = 0.029)  2) Group differences in Categories achieved (*p* = 0.051) and perseverative errors  (*p* = 0.053) of the WCST, with ODT performing slightly better  -when eliminating patients with adverse events, the differences on the WCST and phonemic fluency were not significant  3) Both groups performed worse on Purdue Pegboard  (*p* = 0.0001)  - Statistics performed between groups using mixed effect models and autoregressive covariance to account for repeated measures, *p*-value < 0.05 significant |
| Merola et al.,  2014 | STN | 134 Normal Cognition | Retrospective, observational study | Baseline, 1, 3, 5, > 5 years in 4 conditions | 40 Mild cognitively impaired with DBS | PM47, BWR, CBT, PAL (WMS), TMT-B, PVF, CVF | 1) Significant difference between normal cognition and MCI patients in PVF at 1 year, with normal cognition performing worse (*p* = 0.002)  2) Estimated time to dementia was significantly different between both groups (*p* = 0.028) (Normal cognition – 11.08 years vs. MCI – 6.03 years)  - Statistics performed within groups using Wilcoxon and Friedman tests, *p*-value < 0.05 significant |
| Merola et al.,  2014 | STN | 19 | Retrospective study | Baseline and on average 6 years (4-10 years) (ON/ON) | 16 PD drug therapy (4-11 years) | PM47, BWR, CBT, PAL (WMS), TMT-B, Nelson-MCST categories and perseveration, PVF, CVF | STN vs Control  1) Decline in phonemic verbal fluency (*p* = 0.023)  Both groups vs baseline  1) Worsening in PM47 (BMT: *p* = 0.05; STN: *p* = 0.007)  2) BWR showed trend towards worsening, statistically significant only in STN (*p* = 0.033)  3) MCST perseverative significantly declined in both groups (BMT: *p* = 0.035; STN: *p* = 0.05), but TMT-B (*p* = 0.006), MCST categories (*p* = 0.03), PVF (*p* = 0.001), and CVF (*p* = 0.031) declined in STN  - Statistics performed within groups using Wilcoxon rank sum and between groups using Mann-Whitney U tests, *p*-value < 0.05 significant |
| Sáez-Zea et al.,  2012 | STN | 9 bilateral | Prospective, non-randomized, controlled study | Baseline and 6 months | 12 PD medical therapy | WMS-III-R, WAIS-III, TMT, Stroop Test, The ‘A’ Test, AVLT, rhythmic sequences, motor inhibition, Luria’s fist edge palm test, BNT, SVF, PVF, bimanual praxis, two and three dimensional construction, WCST, arithmetic reasoning, copy and maintain alternating patterns | Whole sample vs baseline  1) Worsening on PVF (*p* = 0.007), time to perform TMT-B (*p* = 0.010), Digit Symbol (WAIS) (*p* = 0.013), and the color-naming of Stroop (*p* = 0.016)  2) Trend for slight worsening in PVF and color-naming for Stroop in STN group  3) Trend for slight worsening in TMT-B in controls  - Statistics performed with group using Wilcoxon signed-rank test and between groups using Mann-Whitney U tests, *p*-value < 0.05 significant |
| Marshall et al.,  2012 | STN | 23 | Investigational study | Baseline and 6 months | 20 PD patients | SDMT, TMT, Digit Span, BNT, RAVLT, WCST, SCWT, VF (cued/un-cued) | STN vs controls  1) Significant decline on cued phonemic/phonemic alternating fluency task in DBS compared to controls (*p* = 0.005)  2) Significant decline on phonemic/semantic un-cued in STN patients compared to controls (*p* = 0.04)  - Statistics performed using ANOVA or ANCOVA, *p*-value < 0.05 significant |
| Smeding et al.,  2011 | STN | 105 bilateral | Outcomes study | Baseline and 12 months | 40 PD patients | MDRS, CVF, COWAT, Alternating VF, PASAT, DART, RAVLT, GITSVR (Groningen Intelligence Subtest Visuospatial reasoning), SCWT, OMO (odd man out test), TMT-A, TMT-B, BNT (alternate forms to minimize practice efforts) | 12 months STN vs Control  1) Significant decline on all verbal fluency tasks (CVF: *p* = 0.005, *d* = -0.6; LVF: *p* < 0.001, *d* = -0.8; AVF: *p* = 0.001, *d* = -0.6), MDRS (*p* = 0.001, *d* = -0.5), delayed recall of RAVLT (*p* < 0.001, *d* = -0.6), Stroop Color (*p* = 0.01, *d* = -0.5), Stroop Color-Word Card (*p* = 0.01, *d* = -0.5), immediate recall of RAVLT (*p* = 0.004, *d* = -0.4), Stroop Word Card (*p* = 0.002, *d* = -0.5), and visuospatial reasoning (*p* = 0.02, *d* = -0.3)  - Statistics performed between groups using ANOVA and effect sizes using Cohen-*d*, *p*-value < 0.05 significant |
| Castelli et al.,  2010 | STN | 27 bilateral | Prospective, controlled trial | Baseline (ON/-) and 1 year (ON/ON) | 31 PD optimal medical treatment | RCM, BWR, CBT, PAL, TMT-B, NCST, PVF, CVF (two parallel forms for all memory tests were used) | DBS vs Control at 12 months  1) Significant decline in PVF  (*p* = 0.002)  - Statistics performed using MANCOVAs, *p*-value < 0.05 significant |
| Zangaglia et al.,  2009 | STN | 32 | Long-term controlled study | Baseline (ON/-), 1, 6, 12, 24, and 36 months (ON/ON) | 33 PD patients | MMSE, Logical memory task, Verbal span, Digit span, CBT, WCST, RPM-47, VF | Month 1 vs baseline  1) Significant worsening on WCST (*p* < 0.001), RM47 (*p* < 0.001), and PVF (*p* < 0.001) in STN group  6 months  1) No differences between baseline  36 months  1) Significant decline in MMSE within DBS group (*p* = 0.05)  2) Trends for increase in verbal span, digit span, CBT, Logical memory task in DBS group  3) Significant decline in control group on WCST (*p* = 0.001) and MMSE (*p* < 0.05)  Between groups  1) Controls had significantly higher verbal fluency scores (*p* = 0.014)  - Statistics performed using ANOVAs (*p*-value < 0.05 significant) with post-hoc Bonferroni comparison (*p* < 0.01 significant), within group differences using Wilcoxon signed-rank sum (*p* < 0.05) |
| Witt et al.,  2008 | STN | 60 bilateral | Randomized, multi-center study | Baseline (ON/-) and 6 months (ON/ON) | 63 PD best medical therapy | MDRS, German version of RAVLT, WAIS Digit Span, Benton visual retention test administration, Stroop Interference Test, SVF, PVF, | DBS group compared to BMT  1) Significantly decline in SVF (*p* = 0.03), PVF (*p* = 0.02), and MDRS initiation/preservation  (*p* = 0.02)  2) Decline in reading time under Stroop interference (*p* = 0.001)  3) Higher error rate on Stroop interference (*p* = 0.001)  - Statistics performed within group using Mann-Whitney U and Spearman’s correlation (*p* < 0.05 significant) and between groups with Cohen-*d* |
| Williams et al.,  2011 | STN | 19 bilateral | Outcomes study | Baseline, 6 months, and 2 years | 18 PD patients | MMSE, DRS, RAVLT-total, RAVLT-short term memory, RAVLT-long term memory, BVMT-R-total, BVMT-R delay, SDMT, Trials A, WAIS Digit Span, Stroop Word, COWAT, SVF, BNT, Trails B, WCST, Stroop Color-Word, Clock drawing | DBS vs controls  1) Greater decline on BVMT-R delayed recall (*p* = 0.03)  - RCI indicated that 47% of DBS patients had a reliable decline compared to 25% of PD  Compared to baseline  1) Greater decline on SDMT  (*p* = 0.03)  - STN patients declined more (*p* = 0.001) compared to controls (*p* = 0.05)  2) Greater decline on LVF  (*p* = 0.01) and SVF (*p* = 0.04)  - STN patients declined more on LVF (*p* = 0.004) and on SVF (*p* = 0.007)  3) Trend for decline on Stroop Color-Word (*p* = 0.07)  - STN patients showing a greater decline (*p* = 0.08)  4) No patient in DBS group impaired on clock drawing, whereas control group was impaired (*p* = 0.04)  - Statistics performed using ANOVAs and ANCOVAs (*p* ≤ 0.05 significant) and effect sizes using partial eta-squared |
| York et al.,  2008 | STN | 23 bilateral | Outcomes study | Baseline and 6 months | 27 PD patients | MMSE, MDRS, SDMT, TMT-A, TMT-B, DS, VF, SVF, BNT, RAVLT, BVMT, WCST, SCWT, Clock drawing | DBS vs controls  1) Verbal delayed recall (*p* = 0.003)  Group declines  1) DBS and PD groups demonstrated declines on SVF (*p* < 0.001), Stroop Color (*p* < 0.001), Digit Span (*p* < 0.008), Trails B (*p* < 0.002), and WCST loss of set (*p* < 0.001)  2) DBS patients showed a trend in declines on Stroop Word (*p* < 0.05) and SDMT (*p* < 0.04)  - Statistics performed using ANOVAs and ANCOVAs (*p* < 0.01 significant) |
| Smeding et al.,  2006 | STN | 99 bilateral | Controlled study | Baseline and 6 months | 36 PD  patients | DRS, CVF, COWAT, Alternating VF, DART, PASAT, AVLT, Groningen Intelligence Test (visuospatial reasoning), SCWT, OMO, TMT-A, TMT-B, BNT | Baseline  1) STN group had a lower score on Odd Man Out  2) Control group lower score on delayed verbal recall  Follow-up  1) STN group performed worse on all measures of verbal fluency (CVF: *p* = 0.000, LVF: *p* = 0.01, AVF: *p* = 0.000) compared to control  2) STN demonstrated a decline in the subtests Attention and Initiation/Preservation of DRS, on delayed recall of AVLT (*p* = 0.02), SCWT (*p* = 0.000), and Stroop color (*p* = 0.000) compared to control  3) Correlation was found between decreases on the DRS and AVLT and low levodopa at baseline  - Statistics performed using Mann-Whitney U (*p* < 0.05 significant) and effect sizes using Cohen’s *d,* Pearson’s *r* for associations |
| Alegret et al.,  2004 | STN | 9 bilateral | Controlled study | Baseline, 6 and 12 months | 7 PD apomorphine patients | RAVLT, SCWT, JLOT, TMT, PVF, MMSE | 6-month  1) Worsening of PVF and Stroop Naming in STN-DBS groups  1 year  1) PVF scores returned to baseline values  2) Still significant worsening of Stroop naming  -Statistics performed using Mann-Whitney U Tests. ANOVA, Pearson’s correlation coefficients (p < 0.05) |
| Gironell et al.,  2003 | STN | 8 bilateral | Controlled, comparative study | Baseline and 6 months | 8 PD pallidotomy patients | DSF, DSB, verbal subtest of WAIS, Sternberg paradigm, BVRT, RAVLT, Rey-Osterrieth Complex Figure, SCWT, TMT, arithmetic subtest of WAIS, WCST, PVF, SVF, Luria motor sequences | 1) Decline in SVF within the STN group (p = 0.005)  -Statistics performed using ANOVA tests (p < 0.05) |
| Moretti et al.,  2003 | STN | 9 bilateral | Comparative study | Baseline, 1, 6, and 12 months | 9 PD  patients | SCWT, Raven Strandard Progressive Matrices, DSB, DSF, retrieval of story, syntactic comprehension test and morphological test, SVF, PVF, BLOT | 1-month DBS vs. control group  1) DBS subjects had an increase in total time taken to execute the Stroop test (p < 0.001)* and number mistakes of the Stroop test (p < 0.01)*  2) Decrease in total words produced in SVF (p < 0.001)*, PVF (p < 0.01)*, and syllabic fluency (p <0.001)*  3) Increase in number of intrusion mistakes (p < 0.001)*  4) Decrease of correct recall compared to baseline (p < 0.05)  6 months DBS vs. control group  1) Significant increase in total time required to execute the Stroop test (p < 0.001)*  2) Significant decrease in correct recall compared to baseline (p < 0.05)  3) Decrease in SVF (p < 0.001)*, in PVF (p < 0.01)*, syllabic fluency (p < 0.001)*  4) Significant increase in number of intrusion mistakes in syllabic fluency (p < 0.001)*  12 months DBS vs. control group  1) Significant increase in total time on Stroop (p < 0.001)* and number of mistakes (p < 0.01)*  2) Decrease in total number of mistakes in syntactic comprehension tasks (p < 0.01)*, SVF (p < 0.001)*, PVF (p < 0.01)*, syllabic fluency (p < 0.001)*  3) Increase in number of intrusion mistakes (p < 0.05)*  *Indicate significance difference from baseline as well  -Statistics performed using Wilcoxon tests (p < 0.05) |
| De Gaspari et al.,  2006 | STN | 12 bilateral | Prospective study | Baseline and 12 months | 13 apomorphine patients | MMSE, PVF, CVF, RCM, PWL, CVLT, CBT | 1) Reduction in CVF in DBS group (p = 0.00)  -Statistics performed using Wilcoxon tests with Bonferroni  correction (p < 0.05) |
| Smeding et al.,  2005 | STN | 20 bilateral | Follow-up study | Baseline, 6, and 12 months | 14 unilateral pallidotomy | DRS, CVF, COWAT, Alternating VF, ART, PASAT, AVLT, Groningen Intelligence Test, SCWT, OMO, TMT, BNT | 6 months  1) Increase in number of errors on the SCWT (p = 0.006) and TMT-B (p = 0.02) in the STN group, whereas patients in the pallidotomy group had a decrease in errors  - Increase in errors on SCWT correlated significantly with a lower baseline DRS scores  12 months  1) STN group showed a trend toward decline on the DRS  -significantly correlated with a decrease in CVF  -Statistics performed using Mann-Whitney U tests (p < 0.05) |
| Morrison et al.,  2004 | STN | 17 bilateral | Follow-up study | Baseline and on average 13.3 weeks | 11 PD  patients | DRS, NART, Randt Memory Test (Digit Span, passages, pictures), Brief Test of Attention, HVLT, BNT, PVF, SVF, visual form discrimination test, JLOT, standardized test of direction sense, OMO, SCWT, Alternating VF | 1) DBS subjects declined on the Attention Composite Score (p = 0.03) and Language Composite Score (p = 0.031) when comparing to controls  2) Stim on vs. stim off demonstrated no significant changes in scores  3) DBS subjects declined on Language Composite Score (p = 0.014) and Delayed Recall Composite Score (p = 0.036) when comparing baseline to stim on, whereas controls did not  4) Controls performed better on SCWT-interference than DBS subjects stim-off (p = 0.045)  5) Controls performed better on SCWT-interference than DBS subjects on stim (p = 0.034)  -Statistics performed using MANOVA and ANOVA tests (p < 0.05) |

| **Supplementary Table 5. Parkinson’s Disease without Control Groups Cognitive Outcomes** | | | | | | |
| --- | --- | --- | --- | --- | --- | --- |
| **Author** | **Target** | **N** | **Type of Study** | **Evaluation Time** | **Tests Performed** | **Outcomes** |
| Rizzone et al.,  2014 | STN | 26 | Long-term study | Baseline, 1, 5, and 11 years | MMSE, PM-47, Digit Span Forward, CBT, MWCST, Phonological VF, Digit Span Backwards, RAVLT, PAL, Attentive matrices, SVF | 1) Significant decline in phonological VF  2) Significant decline in CBT forward (short-term memory), immediate and delayed recall on the RAVLT (episodic memory), executive function (WCST), attention (attentive matrices)  - Statistics performed using Friedman ANOVA with Wilcoxon matched pair for post-hoc and between group comparisons using Mann-Whitney U (*p* < 0.05 significant)  -Individual p-values not reported |
| Tang et al.,  2015 | STN | 27 bilateral | Follow-up study | Baseline, 6, 12 months (ON/ON) | MoCA, CVLT, BVRT, BNT, HVOT, Digit Span, Stroop Test, SVF, CVF | 1) Total CVF significantly declined (*p* = 0.014)  -First significant reduction happened from 0 to 6 months (*p* = 0.011)  2) Significant reduction of animal verbal fluency (*p* = 0.006)  -Happened within first 6 months (*p* = 0.009)  3) Significant improvement on immediate recall of CVLT (*p* = 0.028) (first at 6 months – *p* = 0.006)  - Statistics performed using Friedman’s and then post-hoc analyses with Wilcoxon signed-rank tests with Bonferroni adjustment (*p* < 0.05 significant for ad-hoc and *p* < 0.017 for post-hoc) |
| Aono et al.,  2014 | STN | 13 | Investigational Study | Baseline, 1 month (ON/ON), and 6 moths (ON/ON) | MMSE, WCST, VF | Baseline vs 1 month  1) WCST total error (*p* = 0.005) and semantic VF (*p* < 0.001) were worse  -diminished at 6 months  2) Trends toward decrease in phonetic verbal fluency (*p* = 0.015)  - Statistics performed using ANOVA with Bonferroni adjustment (*p* < 0.01 significant); post-hoc comparisons using Dunnett |
| Janssen et al.,  2014 | STN | 26 | Observational, cohort study | Baseline, 3 months, 1, 5, and 10 years (ON/ON) | MMSE, CVLT, COWAT, SCWT | 1) SCWT (*p* = 0.001) and VF (CVF: *p* = 0.003, LVF: *p* = 0.041) showed a decrease after surgery 1 and 5 years  - Statistics performed using Friedman test and Wilcoxon signed-rank as post-hoc (*p* < 0.05 significant) |
| Harati et al.,  2013 | STN | 20 bilateral | Follow-up study | Baseline and between 12 to 18 months (ON/ON) | MMSE, PANDA, VLMT, WMS, CDT, LPS, PVF, SVF | 1) Decline in digit span forward (*p* < 0.01) and backward (*p* < 0.05)  2) Decline in VLMT trial-5 (attention) (*p* < 0.01)  3) Decline in VLMT interference (episodic memory) (*p* < 0.01)  4) Decline in PVF (*p* < 0.001) and SVF (*p* < 0.05)  - Statistics performed using Wilcoxon rank sum (*p* < 0.05 significant) |
| Yágüez et al.,  2013 | STN | 30  bilateral | Investigational Study | Baseline and on average 9.4 months | WAIS Vocabulary, Similarities, Arithmetic, Digit Span, Picture Completion, Block Design, Matrix Reasoning, and Picture Arrangement, Recognition memory tests, Birt memory and information processing battery, GNT, Incomplete Letters and Object Decision tasks from the Visual Object and Space Perception Battery, Hayling Sentence Completion Test, Brixton Spatial Anticipation Test, PVF | 1) Decline in Verbal IQ (small effect) (*p* = 0.015, *d* = 0.19)  2) Decline in Full Scale IQ (medium effect) (*p* = 0.034, *d* = 0.43)  3) Decline in immediate story recall (large) ( *p* = 0.000, *d* = 0.75), delayed story recall (medium) (*p* = 0.015, *d* = 0.50), and list learning (medium) (*p* = 0.003, *d* = 0.46)  4) Decline in verbal fluency (medium – even with parallel versions) (*p* = 0.003, *d* = 0.53)  5) Individual Brixton Tests declined most often  - Statistics performed using paired *t*-tests (*p* < 0.05 significant) and Cohen’s *d* for effect sizes |
| Yamanaka et al.,  2012 | STN | 30 | Investigational study | Baseline (ON/-), 1 and 12 months (ON/ON) | MMSE, RCM, Digit Span Forward and Backward, TMT-B, TMT-A, SVF, Frontal Assessment Battery (FAB), SCWT, PVF | After 1 month  1) TMT-B (*p* < 0.01), TMT (B/A) (*p* < 0.01), SCWT (*p* < 0.01), SVF (*p* < 0.01), PVF (*p* < 0.01) declined  At 12 months  1) MMSE significantly better at 12 months than baseline (*p* < 0.05)  2) FAB significantly improved at 12 months (*p* < 0.01)  3) Significant decline in SCWT (*p* < 0.01) and SVF (*p* < 0.05), but they were still better than 1-month scores  - Statistics performed using ANOVA for repeated measures and Bonferroni corrections (*p* < 0.05 significant) |
| Heluani et al.,  2012 | STN | 20 | Outcomes study | Baseline (ON/-) and 6 months (ON/ON) | MMSE, Subtests of vocab and matrix reasoning from WAIS, Digit span forward and backward, HVLT, BVMT, PVF, SVF (animals), MWCST, SDMT, BNT, Stroop test, TMT-A/B, Spatial construction-subtest block design | 1) No significant differences  - Trend for decline on PVF (*p* = 0.081)  - Statistics performed using paired *t*-tests (*p* < 0.05 significant) |
| Zibetti et al.,  2011 | STN | 14 bilateral | Long-term outcomes study | Baseline, 1, 5, and > 9 years (ON/ON) | PM47, BWR, CBT, PAL, TMT-B, MCST, PVF, CVF | 1) BWR significantly worsened at > 9 years compared to baseline, 1, and 5 years  2) TMT-B improved 1 year after surgery and then worsened at 9 years  3) Total and perseverative errors on the MCST increased significantly 5 and 9 years after surgery  4) Progressive and significant worsening of PVF and CVF  - Statistics performed using Friedman and Wilcoxon signed-rank tests (*p* < 0.05 significant)  - Individual *p*-values not reported |
| Fasano et al.,  2010 | STN | 20 | Long-term outcomes study | Baseline, 5, and 8 years (ON/ON) | MMSE, CBT, RAVLT, PM-47, PVF, MWCST | Baseline vs. 5 years  1) Significant decline in LVF (*p* < 0.05), PM47 (*p* < 0.05), and delayed recall of the RAVLT (*p* < 0.05)  Baseline vs. 8 years  1) Significant decline in LVF (*p* < 0.01), PM47, immediate and delated recall of RAVLT (*p* < 0.01), and number of correct criteria of the MWCST (*p* < 0.05)  8 vs 5 years  1) Significant decline in immediate recall of the RAVLT (*p* < 0.05)  - Statistics performed using Wilcoxon signed-rank tests (*p* < 0.05 significant) |
| Denheyer et al.,  2009 | STN | 23 bilateral | Short-term outcomes study | Baseline and 1 year | PVF, SVF, WCST, NART | 1) Decline in SVF (*p* = 0.04) and PVF  (*p* = 0.03)  - When corrected for multiple comparisons, only changes in levodopa and PVF remained significant  - Statistics performed using Wilcoxon signed-rank tests or paired *t*-tests (*p* < 0.05 significant) |
| Heo et al.,  2008 | STN | 46 bilateral | Short-term outcomes study | Baseline (ON/-), 6 months and 1 year (ON/ON) | TMT, BNT, Rey-Kim Memory battery, Grooved pegboard (sensory-motor coordination), Stroop Test, SVF, PVF, WCST, MMSE | 1) Significant differences between baseline and post-surgical in verbal memory (recognition at 6 months (*p* < 0.01) and delayed recall (*p* < 0.05) and recognition at 1 year (*p* < 0.05))  2) Significant difference on the Stroop test (6 months only Neutral Word reading (*p* < 0.05), but at 1 year there were declines in all categories: color dot (*p* < 0.01), neutral word (*p* < 0.01), color-word (*p* < 0.05))  3) Significant difference on the word fluency test (difference at 6 months (*p* < 0.05) and 1 year (*p* < 0.05))  - Statistics performed using paired *t*-tests (*p* < 0.05 significant) |
| Higginson et al.,  2008 | STN | 22 bilateral | Short-term outcomes study | Baseline (ON/-), and 6 months (ON/ON) | CVLT, WCST, LVF, SVF, BNT (practice efforts corrected using RCI) | 1) Significant decline on animal fluency (*p* = 0.008), total correct responses on learning trials of the CVLT (*p* = 0.024), and letter fluency (*p* = 0.028)  - Statistics performed using ANOVA (*p* < 0.05 significant) |
| Fraraccio et al.,  2008 | STN | 15 | Outcomes study | Baseline and 15.9 months (4-49 months) ON and OFF stim | WMS, RAVLT, Tower of London, Rey Osterrieth Figure (alternative versions used when possible), Externally ordered working memory task, WAIS-digit span, WCST, Stroop, SDMT, WASI-Digit Span (Forward), HVOT, Rey Figure/Taylor figure, BNT, COWAT, Sequential and simple tapping, grooved pegboard, MMSE | 1) On grooved pegboard, time interval for peg insertion was significantly improved by stimulation when using dominant (*p* < 0.005) or non-dominant hand (*p* < 0.0001)  2) On sequential tapping, stimulation significantly improved motor speed for non-dominant hand (*p* < 0.038)  3) On Stroop-interference, a significant higher sensitivity was observed post-op (*p* < 0.003)  4) Significant reduction in word reading (processing speed) (*p* < 0.004)  - Statistics performed using paired *t*-tests (*p* < 0.05 significant) |
| Kishore et al.,  2010 | STN | 45 bilateral | Long-term, open-label study | Baseline (ON/-), 1, 3, and 5 years (ON/ON) | Digit Span, VF, TMT-A/B, WCST, Praxis (Object assembly, block design), SVF, CVF, 12-item line drawing, WMS, JLOT, visual object, space perception battery, MMSE | 1) No significant decline in any of the cognitive spheres  - Baseline: 1 person had a verbal fluency 1 SD below norm  - 1-year: 6 people  - 5-year: 9 additional patients  - Statistics performed using ANOVA, post-hoc comparisons were made using *t*-tests or Wilcoxon signed-rank (*p* < 0.005 significant) |
| Lefaucheur et al., 2012 | STN | 26 | Short-term outcomes study | Baseline (ON/-), 3 days (OFF/OFF), 10 days (ON/OFF) and 6 months (ON/ON) | Verbal fluency and Stroop test | 1) Number of words (*p* < 0.01) and switches (*p* < 0.01) were significantly reduced immediately in post-op, but there was reliable improvement from day 3 to 6 months (*p* < 0.01)  2) Time to read color (*p* < 0.01) and word (*p* < 0.01) increased significantly immediately post-op and correlated with semantic fluency at day 3 and day 10 and phonemic fluency at day 10  - Statistics performed using ANOVA (*p* < 0.05 significant); Spearman rank correlation used to determine relationships |
| Bonenfant et al.,  2017 | GPi | 25 | Follow-up study | Baseline, 1 and 3 years | MDRS, TMT, WCST, Stroop test, SVF, PVF | 3-years  1) Significant decline in MDRS (*p* = 0.049), Stroop Color-Word (*p* = 0.01), Stroop Interference (*p* = 0.01), and TMT A-B (*p* = 0.01)  2) SVF and PVF remained stable as well as WCST  - Statistics performed using Wilcoxon signed-rank (*p* ≤ 0.05 significant) |
| Tröster et al.,  1997 | VIM | 9 | Follow-up study | Baseline and 4 months | MDRS, Brief test of attention, WCST, COWAT, BNT, HVOT, JLOT, facial recognition test, CVLT, WMS (logical memory and figural memory), Kaufman Adolescent and Adult Intelligence Test Famous Faces subtest | 1) Significant improvement in delayed recognition of CVLT and delayed recall of WMS-Logical Memory  - Statistics performed using Wilcoxon signed-rank (*p* ≤ 0.05 significant) |
| Vingerhoets et al., 1999 | GPi | 20 | Follow-up study | Baseline and 3 months | RAVLT (alternate versions), Benton visual retention test, Purdue pegboard test, Visual reaction time, Visual object and space perception battery (number location and cube analysis), Money’s standardized road map test, SCWT, COWAT, WCST, NART | 1) Patients who performed cognitively worse after stimulation were significantly older at the time of surgery (*p* = 0.04) and received higher doses of levodopa (*p* = 0.01  2) All patients with cognitive decline (n = 6) all had implants in the left hemisphere (*p* < 0.05)  3) No significant changes in cognitive scores  - Statistics performed using repeated MANOVAs (*p* ≤ 0.05 significant), for individual outcomes a cognitive impairment index was calculated |
| Tröster et al.,  1997 | GPi | 9 unilateral | Follow-up study | Baseline and 3 months | DRS, Brief Test of Attention, Stroop Neuropsychological Screening Test, WMS (Mental Control, Figural Memory, Logical Memory I and II, CVLT), WCST, Cognitive Estimation Test, COWAT, CVF, BNT, Facial Recognition Test, HVOT, Grooved Pegboard, Famous Faces | 1) Significant decline in SVF (p = 0.05) and construction scores on the DRS (p = 0.05)  - Statistics performed using Wilcoxon’s signed rank test (*p* ≤ 0.05 significant), for individual outcomes a cognitive impairment index was calculated |
| Fields et al.,  1999 | GPi | 6 bilateral | Follow-up study | Baseline, 2 months post-op (unilateral), 3 months post-op (bilateral) | DRS, Brief test of attention, SCWT, WCST, LVF, SVF, BNT, Grooved pegboard, facial recognition, HVLT, WMS | 1) Number of perseverative errors on WCST decreased at unilateral surgery, but not bilateral (p < 0.05)  2) Improvement in long-delay free recall of CVLT and delayed recall of LMII of WMS at bilateral (p < 0.05)  3) Construction scores from DRS at both unilateral (p < 0.07) and bilateral (p < 0.06) tended to decline  4) Number of failures to maintain set from baseline to bilateral (p = 0.08) and scores on Cognitive Estimation Test increased from unilateral (p < 0.06) and bilateral (p < 0.07)  5) SVF trended toward decline (p < 0.08) but only after the first procedure  6) LMII scores tended to increase (p < 0.07) after unilateral  -Statistics performed using Wilcoxon signed-rank tests (p < 0.05 significant) |
| Alegret et al.,  2001 | STN | 15  bilateral | Follow-up study | Baseline and 3 months | RAVLT, BLOT, TMT, PVF, SVF, SCWT | 1) Deterioration in performance on the RAVLT (p = 0.02), BLOT (p = 0.03), PVF (p = 0.005), SVF (p = 0.008), and Stroop color (p = 0.04)  2) Improvement in TMT-B (p = 0.03)  3) Rate of learning decreased after DBS according to RAVLT  -Statistics using paired-sample t-tests and repeated measures ANOVA (p < 0.05) |
| Castelli et al.,  2006 | STN | 65 bilateral | Follow-up study | Baseline and 15 months | PM-47, BWR, CBT, PAL, WMS, TMT-B, MCST, WCST | 1) Lower number of total errors (p < 0.02) and preservative errors (p < 0.04) in the MCST  2) Decline in PVF (p < 0.001) and CVF (p < 0.005)  -Statistics performed using paired t-tests (p < 0.05) |
| Saint-Cyr et al.,  2000 | STN | 11 bilateral | Follow-up study | Baseline, 3- 6, and 9-12 months | DSF, DSB, PASAT, TMT, Purdue Pegboard, Finger tapping, Conditional Associative Learning Test, PVF, SVF, CVLT, Battery of Memory Efficiency | 1) Reduced finger tapping with and without divided attention, slower set alternation, decreased LVF, and impaired verbal and nonverbal memory at 3-6 month post-op  2) Decline in working memory across entire group and elderly subgroup was seen at 9-12 month follow-up  3) Patients had more difficulties with bimanual coordination post-op, and elderly subjects were more vulnerable  4) Increase in time to complete TMT-B and increased problems on Conditional Associative Learning Test at 9-12 months  5) Decline on PVF and CVF and did not recover at 1 year or at longer follow-ups, specifically declines on switching  6) Significant declines on the long delay free recall and long delay cued recall at 3-6 months, and recovered slightly. In elderly subgroup, more early difficulty in verbal learning, especially on short-delay free recall score  7) Total scores on 7 visual subtests of the BEM declined at 3-6 month follow-up but resolved at 9-12 month evaluation  -Statistics performed using paired t-tests and repeated measures ANOVA |
| Contarino et al.,  2007 | STN | 11 bilateral | Follow-up study | Baseline, 1, and 5 years | MMSE, DSF, DSB, CBT, RAVLT, PM47, LVF, WCST | One-year  1) Decline on LVF (p = 0.045)  2) Improvement on MMSE (p = 0.009)  Five-year  1) Decline on LVF (p = 0.007) and PM47 (p = 0.009)  2) Trend towards decline on delayed recall of the RAVLT (p = 0.075) and Corsi’s span backward (p = 0.091)  1-year vs 5-year  1) Decline on LVF (p = 0.005), MMSE (p = 0.008), and Corsi’s span backward (p = 0.028)  -Statistics performed using Wilcoxon’s signed-rank test (p < 0.05) |
| Dujardin et al.,  2001 | STN | 9 bilateral | Follow-up study | Baseline, 3, and 12 months | DSB, DSF, Corsi spatial span test, Belleville et al. word span test, Belleville alphabetic span test, Grober and Buschke word list learning and recall test, Baddeley’s doors test, Tower of London test, VF, spatial sequences generation task, SCWT, TMT, WCST | 3 months (n = 9)  1) Improvement of DSF (p = 0.03) and reduction in response time on an information processing speed task (p = 0.027)  2) Reduction on the number of words in the Grober and Buschke delayed free recall (p = 0.01) and number of words on CVF (p = 0.042)  1 year (n = 9)  1) A trend toward shorter simple reaction time  2) Trend for the number of words named in the CVF (p = 0.040)  3) Interference index of the Stroop task significantly higher (p = 0.02) and trend towards increase in TMT-B (p = 0.04)  4) Number of errors on WCST showed a trend toward increase (p = 0.05)  -Statistics performed using Wilcoxon’s signed-rank test (p < 0.025) |
| Funkiewiez et al.,  2004 | STN | 77 bilateral | Follow-up study | Baseline, 1, and 3 years | DRS, WCST, CVF, LVF, graphic and motor series, Grober and Buschke test | 1) Decrease in CVF at one year (p < 0.001) and 3 years (p = 0.001)  2) Number of words (CVF and LVF) decreased at one (p = 0.0011) and 3 years (p = 0.0006)  - Statistics performed using an ANOVA and a Bonferroni correction (p < 0.01) |
| Slowinski et al.,  2007 | STN | 24 unilateral | Follow-up study | Baseline, 1, 3, and 12 months | DRS, Digit Span (WAIS), COWAT, SVF, JLOT, TMT-A, TMT-B, HVLT | 1) Improvement in TMT-B (p < 0.05), which is a visuomotor task requiring mental tracing and divided attention  2) Nonsignificant trend of decline on PVF (p = 0.053)  - Statistics performed using two-tailed paired t-test or Wilcoxon signed-rank test (p < 0.05) |
| Perozzo et al.,  2001 | STN | 20 bilateral | Follow-up study | Baseline and 6 months | PM47, BWR, CBT, PAL, spatial learning, Rivermead behavioral memory test, visual search, simple and choice reaction time, WCST (simplified), TMT-B, CVF, LVF | 1) No cognitive declines after surgery  - Statistics performed using ANOVA for repeated measures (p < 0.05) |

| **Supplementary Table 6. Parkinson’s Disease Correlation Studies Cognitive Outcomes** | | | | | | | |
| --- | --- | --- | --- | --- | --- | --- | --- |
| **Author** | **Target** | **N** | **Type of Study** | **Evaluation Time** | **Control Group** | **Tests Performed** | **Outcomes\Correlations** |
| Smith et al.,  2014 | STN | 50 bilateral | Retrospective study focused on number of MER tracks | Baseline and 6-24 months (ON/ON) | NA | MDRS, TMT-B, TMT-A, RAVLT, PVF, CVF, subtests from WAIS | 1) Significant decline in PVF (*p* = 0.003)  - moderately correlated with age at surgery (r = 0.38, p = 0.07), but not PD duration (r = 0.001, p = 1.0), baseline global cognition (r = -0.17, p = 0.41), number of MER passes (r = -0.02, p = 0.94), or stimulation parameters  - Statistics performed using pairwise correlation analysis and *t*-test (*p* ≤ 0.05 significant) |
| Mikos et al.,  2011 | STN | 17  (9 left, 8 right) | Correlation study (Data from prospective randomized trial) focused on VTA | Baseline and 7 months (OFF stim, ON stim: optimal, ventral, dorsal) | NA | Verbal fluency (letter and semantic) – alternate forms were employed to minimize practice efforts | 1) With optimal stimulation, there was a positive correlation between VTA inside the STN and letter fluency change scores (*r* = 0.44, *p* = 0.10)  - larger VTA was associated with improved verbal fluency scores  - more VTA inside STN associated with better fluency performance relative to OFF stimulation  2) With optimal stimulation, positive correlation with letter fluency change scores and total VTA and the percent VTA overlap with STN (*r* = 0.50, *p* = 0.06)  3) With ventral stim, there was a negative correlation between VTA inside the STN and letter fluency change scores (*r* = -0.60, *p* = 0.024)  - more VTA inside STN was associated with worse letter fluency performance  - larger volume of VTA inside STN was associated with worse letter fluency performance relative to OFF stimulation condition  - Relationships 1-3 were not found with category fluency  4) No differences in verbal fluency scores among the three electrode contacts (optimal, dorsal, ventral)  - Statistics performed using ANOVA and Spearman’s correlation coefficient (*p* < 0.05 significant) |
| Dietz et al.,  2013 | GPi | 14 (6 left, 8 right) | Correlation study (Data from prospective randomized trial) focused on VTA | Baseline and 7 months (OFF stim, ON stim: optimal, ventral, dorsal) | NA | Verbal fluency (letter and semantic) – alternate forms were employed to minimize practice efforts | 1) No significant relationships between magnitude and location of VTA and fluency performance relative to OFF stim condition  - Statistics performed using Wilcoxon signed-tank tests, Friedman’s test and Spearman’s correlation coefficient (*p* < 0.05 significant) |
| Blume et al.,  2017 | STN | 40 bilateral | Retrospective study focused on white matter lesions | Baseline (ON/-), and 3 years | NA | TMT A/B, SDMT, Similarities Test, SVF, COWAT, Rey complex figure retention, logical memory scale 1 and 2, Rey complex figure copy, block design test | 1) Significant decline on SVF (*p* = 0.00001), TMT-A (*p* = 0.017), and block design test (*p* = 0.011) compared to baseline  -15 of the patients at 3 years fulfilled the criteria for PD-MCI or PD-D  - Only difference between those that developed PD-D and non-demented were age and occurrence of hallucinations at baseline  -White matter lesions (WML) were significantly associated with age (*p* = 0.03; R^2^ = 0.12) and one or more cardiovascular risk factors (*p* = 0.01)  -Patients who developed PD-D had a significantly higher volume of WML at baseline compared to non-demented (*p* = 0.009)  2) Decline of Cognitive Composite Score was significantly correlated to the WML volume after correction for age (*p* = 0.006; R^2^ = 0.40)  - Statistics performed between groups using *t*-test and Mann-Whitney U and correlations using Pearson correlation coefficient (*p* < 0.05 significant) |
| Isler et al.,  2016 | STN | 30 bilateral | Prognostic study investigated caudate penetration | Baseline (ON/-), 3, and 12 months (ON/ON) | NA | LVF, CVF, SCWT, RAVLT, TMT A and B | 3 months  1) TMT-B showed an interaction within groups over time showing a higher decrease in the caudate group vs. non-caudate group (*p* = 0.002)  2) Verbal fluency reached the main effect of time (LVF: *p* = 0.016; CVF: *p* = 0.00), but not different between groups  3) 25% of patients in the caudate group significantly declined on the Stroop interference vs none in the non-caudate group (individual change is higher than 1 SD at baseline)  12 months  1) TMT-B showed main effect of time with a significant decline in both groups (*p* = 0.000)  - Trend for a higher decrease in caudate group (*p* = 0.070)  2) Verbal fluency reached a main effect of time in total sample (LVF: *p* = 0.012; CVF: *p* = 0.000)  - Statistics performed between follow-ups using repeated measures ANOVA and partial eta-squared for effect size (*p* < 0.05 significant) |
| Witt et al.,  2013 | STN | 31 (30 bilateral) | Retrospective study focusing on electrode trajectory | Baseline and 6 months (ON/ON) | NA | MDRS, Digit Span Backward (WMS), LVF, SVF, Stroop (parallel versions used) | 1) Significant reduction in verbal fluency (SVF: *p* = 0.02) compared to BMT  - Patients who declined in MDRS (*p* = 0.02) and digit span backwards (*p* < 0.04) had a more medially located electrode, which passed through the caudate – For every 0.1 ml volume penetrated by the electrode within the caudate, risk in declining in global cognitive performance increased by a factor of 37 for MDRS and 8.8 for digit span  - Stroop task performance was significantly correlated with cortical entry points and patients who decline in Stroop had an entry point outside the area of stable performers  - Statistics performed using *t*-tests or Mann-Whitney-U Tests and a permutation test (*p* < 0.05 significant) |
| York et al.,  2009 | STN | 17 bilateral | Retrospective study focusing on electrode trajectory | Baseline and 6 months (ON stim) | NA | RAVLT, VF (alternate forms), MMSE, MDRS, SVF | 1) Significant decline in RAVLT total (*p* = 0.05), RAVLT long-term memory (*p* = 0.02), Verbal fluency (*p* = 0.05), semantic fluency (*p* = 0.001) and MMSE (*p* = 0.04)  -patients who ventricles were pierced had a decline on long-term memory and verbal fluency (not caudate) (*p* = 0.06)  2) Declines in MMSE were related to electrodes more laterally places in either hemisphere, particularly when active electrodes were posterior-lateral  3) Declines in DRS were related to electrodes more superiorly located in the left hemisphere  4) Declines at 6 months in verbal learning had electrodes closer to the STN and more superiorly in the left hemisphere, however in the right the electrodes located more in the lateral were related to declines  5) Declines in long-term verbal memory were found for electrodes more posterior-lateral in the left hemisphere  6) Declines in verbal fluency scores were more variable with correlations found between change scores and electrodes located in the lateral and superior directions  7) Semantic fluency was correlated with right hemisphere electrodes placed more superiorly  - Statistics performed using ANOVA for differences following surgery and correlation analyses (*p* < 0.05 significant) |
| Le Goff et al.,  2015 | STN | 59 bilateral | Retrospective analysis focusing on electrode trajectory | Baseline (-/ON), and 6 months (ON/ON) | NA | MDRS, SVF, PVF, Stroop | 1) Significant reduction in SVF (45 patients declined more than 5%, while 15 remained stable) (*p* < 0.001)  -Patients who declined more had a trajectory with a more anterior cortical entry point passing less frequently through the thalamus in the left hemisphere (*p* = 0.03)  -Within group that declined, they had a significant increase in both time on word and color on Stroop (*p* < 0.001) and decrease in MDRS (*p* = 0.02) compared while both remained stable in those that did not decline (*p* > 0.05)  2) Significant reduction of PVF (45 patients declined more than 5%, while 15 remained stable) (*p* < 0.05)  -No significant difference in trajectories  -Within group that declined, they had a significant increase in both time on word and color on Stroop (*p* < 0.001), when it remained stable in those that did not decline (*p* > 0.05)  - Statistics performed between time points using Wilcoxon signed-rank test and then between groups (decline vs stable) using Mann-Whitney (*p* < 0.05 significant) |
| Floden et al.,  2017 | STN | 46 (23 bilateral, 8 right, 15 left) | Retrospective analysis focusing on contact location | Baseline and 3-29 months after | NA | PVF, SVF, RAVLT, WCST | 1) Significant worsening of both SVF (*p* < 0.001, *d* = 0.84) and PVF (*p* < 0.001, *d* = 0.56)  -Significant relationships between contact location and both semantic and phonemic verbal fluency  -Semantic worsened with more medial contact placement on the left in those with unilateral left and bilateral implants (*r* = −0.38, *p* = 0.048)  -Phonemic fluency worsened with more posterior-left contacts with patients in unilateral left or bilateral implants (*r* = 0.35, *p* = 0.036)  -Patients with right unilateral electrodes had a significant relationship between increased stimulation voltage and worsened single trial learning (*r* = -0.40, *p* = 0.022)  - Statistics performed between time points using Wilcoxon signed-rank test or t-tests and then between groups using Mann-Whitney U-tests and Cohen’s *d*. Pearson’s or Spearman’s were used for correlation analyses. (*p* < 0.05 significant) |
| Cilia et al.,  2007 | STN | 20 bilateral | SPECT imaging study | Baseline and 12 months | 12 PD patients | MMSE, PVF, CVF, WCST, PM47 | 1) Significant decline in CVF (p < 0.01)  -Decline in performance was significantly correlated with hypoperfusion clusters in dorsolateral prefrontal cortex, anterior cingulate cortex, and the ventral part of the caudate nucleus on the left hemisphere and premotor area on the right hemisphere  -Statistics performed using an ANOVA (p < 0.05 after Bonferroni correction) |

| **Supplementary Table 7. Parkinson’s Disease Different DBS Techniques Cognitive Outcomes** | | | | | | | |
| --- | --- | --- | --- | --- | --- | --- | --- |
| **Author** | **Target** | **N** | **Type of Study** | **Evaluation Time** | **Control Group** | **Tests Performed** | **Outcomes** |
| Trӧster et al.,  2017 | STN | 101 bilateral | Randomized, controlled trial testing constant current devices | Baseline, 3 months (active and delayed stim), and 12 months | 35 delayed stimulation | DRS, SCWT, TMT, Delis-Kaplan Executive Function System, WCST, WMS, HVLT, WAIS, BNT | 3 months – STIM Group  1) Significant declines in all parts of the Stroop (*p* < 0.05) and on the letter verbal fluency (*p* < 0.05)  2) Increase in scores for delayed story recall (*p* < 0.05) and immediate and delated recall of pictured scenes (*p* < 0.05)  3 months – Control group  1) Significant decrease in initiation score on the DRS (*p* < 0.05)  2) Whole cohort declined on category and switching fluency  12 months (*p* < 0.05)  1) Decline on WASI Vocabulary (*p* < 0.05)  2) Significant declines on the Stroop word (*p* < 0.05), color (*p* < 0.05), color-word test (*p* < 0.05), verbal fluency (*p* < 0.05)  3) Significant increase on working memory (*p* < 0.05)  - Statistics performed between groups were ANCOVA and within group were *t*-tests. Pearson’s correlation coefficient and regression analyses were used for correlation (*p* < 0.05 significant) |
| Brodsky et al.  2017 | STN or GPi | 18 STN/21 GPi MER | Outcomes study | Baseline and 6 months | 30 DBS using iCT (7 STN/23 GPi) | MDRS, COWAT | 6-month  Category fluency  1) Improvement in category fluency in asleep DBS patients (*p* = 0.022)  2) Decline in category fluency in awake DBS patients (*p* = 0.0021) (significance between both groups as well – *p* = 0.0012)  -Observed even when controlling for target (*p* = 0.0013)  Phonemic fluency  1) Unchanged in asleep (*p* = 0.58) and worsened in awake (*p* = 0.0072) (significant difference between groups – *p* = 0.038)  -Observed even when controlling for target (*p* = 0.038)  2) MDRS stayed stable in both groups (*p* = 0.44)  - Statistics performed were ordinary least squares regression, Bonferroni corrected (*p* < 0.05 significant) |
| Ostrem et al.,  2016 | STN or GPi | 16 STN/4 GPi | Outcomes study | Baseline and 1 year | NA | Delis-Kaplan Executive Function System, SDMT, Rey-Osterrieth Complex Figure, BNT, Animal Verbal Fluency, LVF, MoCA | 1) Mild decline in verbal category fluency in 35% of patients  2) Mild decline in complex figure copy (35%)  3) Mild decline on 1 delayed memory task (30%)  -Values were considered when greater or less than 1 SD from baseline |

| **Supplementary Table 8. Parkinson’s Disease Studies Comparing STN and GPi Cognitive Outcomes** | | | | | | | |
| --- | --- | --- | --- | --- | --- | --- | --- |
| **Author** | **Target** | **N** | **Type of Study** | **Evaluation Time** | **Control Group** | **Tests Performed** | **Outcomes** |
| Odekerken et al.,  2015 | STN/GPi | 58 GPi and 56 STN | Randomized, controlled trial | Baseline (ON/-) and 1 year (ON/ON) | NA | SCWT, TMT-A, MDRS, TMT-B, Letter and number sequencing of WAIS-III, digit span of the WAIS-III, Vienna test system simple and choice reaction tests, WCST, COWAT, SVF, BNT, similarities of WAIS-III, RAVLT, logical memory by Rivermead behavioral memory test, Matrix reasoning by WAIS-III (parallel tests versions were used) | 1-year  1) Stroop word reading (GPi mean [SD] 21.1 [10.4], STN 27.0 [11.3], CI 1.9–10.0), Stroop color naming (GPi 22.6 [9.6], STN 28.1 [8.1], CI 2.1–8.8), TMT-B (GPi  20.7 [12.0], STN 26.1 [14.2], CI 0.5–10.3), and borderline significance on WAIS similarities (GPi 20.1 [2.3], STN 20.8 [1.7], CI 20.01 to 1.5) with STN showing greater decline  - Statistics performed were either *t*-tests or Mann-Whitney U tests, effect sizes were calculated using Cohen *d* (*p*-value < 0.05 significant) |
| Boel et al.,  2016 | STN/GPi | 39 STN and 39 GPi | Randomized, controlled trial | Baseline (ON/-), 1 (ON/ON), 3 years (ON/ON) | NA | SCWT, TMT-A, MDRS, TMT-B, Letter and number sequencing of WAIS-III, digit span of the WAIS-III, Vienna test system simple and choice reaction tests, WCST, COWAT, SVF, BNT, similarities of WAIS-III, RAVLT, logical memory by Rivermead behavioral memory test, Matrix reasoning by WAIS-III (parallel tests versions were used) | 3-year  1) No significant group differences  - Statistics were performed using linear mixed model analyses (*p*-value < 0.05 significant) |
| Rothlind et al.,  2015 | STN/GPi | 84 STN and 80 GPi | Prospective, randomized, controlled trial | Baseline and 6 months (ON/ON) | 116 BMT | WAIS-III (digit symbol, symbol search, digit forwards and backward, letter-number, arithmetic, similarities), TMT-A, TMT-B, Animal naming, Grocery naming, SCWT, PVF, BNT, HVLT (trials 1-3, delayed recall, recognition), BVMT (trials 1-3, delayed recall, discrimination), brief visual memory test, WCST (preservations, total error), Stroop interference (alternate versions used for learning and memory) | STN vs GPi  1) Significant decline on Stroop word reading in STN (*p* = 0.05)  2) Significant decline on HVLT in GPi (*p* = 0.05)  DBS vs BMT  1) Greater decline in WAIS-III-digit symbol, symbol search, letter-number, arithmetic, similarities, Grocery and animal naming, Stroop color naming and word, PVF, BVMT-R delayed recall in DBS group (*p* < 0.05)  -Multiple measures of processing speed and working memory  2) BMT had higher rate of improvement on WCST perseverative responses (*p* < 0.05)  - Statistics performed using multiple *t*-tests (*p*-value < 0.05 significant) |
| Follet et al.,  2010 | STN/GPi | 152 GPi and 147 STN | Prospective, randomized, controlled trial | Baseline, 6, and 24 months | NA | MDRS, WAIS-III (Processing speed, working memory), PVF, SVF, HVLT (learning and memory, delayed recall), BNT, WCST, Brief visual spatial memory (total, delayed recall), finger tapping | 24-month follow-up  1) Significant difference between groups on processing speed index (WAIS) (*p* = 0.03), greater decline in STN  -group differences on digit symbol visuomotor subtest accounted for this effect  -most outcomes reported in Weaver et al.  - Statistics performed using *t*-tests (*p*-value < 0.05 significant) |
| Weaver et al.,  2012 | STN/GPi | 89 GPi and 70 STN | Prospective, randomized, controlled trial | Baseline, 6, 24, and 36 months | NA | MDRS, WAIS-III (Processing speed, working memory), PVF, SVF, HVLT (learning and memory, delayed recall), BNT, WCST, Brief visual spatial memory (total, delayed recall), finger tapping | 1) Significant difference on MDRS  - STN were slightly worse at 6 months vs. GPi with no change (*p* = 0.03)  -further deterioration in both groups, but STN worse (*p* = 0.01)  2) HVLT Trials 1-3 (*p* = 0.01) and HVLT-delayed recall (*p* = 0.004) differed by target  -GPi showing slight worsening at 6 months but was then stable  - STN was unchanged at 6 months, but then worsened at 36 months  3) Trends for decline on WCST (*p* = 0.03), Brief visuospatial memory test total score (*p* = 0.001) and delayed recall (*p* < 0.001)  - Statistics performed using *t*-tests or Fisher’s exact, or mixed-effects models (*p*-value ≤ 0.05 significant) |
| Zahodne et al.,  2009 | STN/GPi | 10 STN (3 right) and 12 GPi (5 right) | Controlled study | Baseline and 12 months | 19 PD controls | SVF (animals), COWAT (letter fluency), Digit span backwards (working memory), BNT, Vocabulary WASI | DBS vs. control at 12 months  1) Decreased COWAT (letter fluency) (*p* = 0.03)  12 months vs. baseline  1) Decrease COWAT (*p* = 0.001) and SVF (*p* = 0.002)  -Trend in GPi for negative correlation with LEED and change in letter fluency (r = 0.55; p = 0.08)  - Statistics performed using ANOVA\ANCOVAS, Bonferroni post-hoc comparisons, and Pearson’s correlation coefficients (*p*-value ≤ 0.05 significant) |
| Mikos et al.,  2010 | STN/GPi | 11 STN (3 right) and 13 GPi (5 right) | Controlled study | Baseline and 12 months | 19 PD controls | HVLT, the immediate and delayed recall trials from the Logical Memory subtest of WMS, TMT, Stroop Test, JLOT, Benton Facial Recognition Test | 12 months vs. baseline  1) Significant decline in visuospatial (Benton Facial, JLOT – *p* = 0.022)  -only scores declined on JLOT (*p* = 0.032)  2) DBS patients scored lower than baseline scores (*p* = 0.041) and PD control’s scores on processing speed (*p* = 0.025)  -found for TMT-A (*p* = 0.018)  3) Both groups declined on Stroop Word Reading Test (*p* = 0.045)  - Statistics performed using MANOVAS followed by ANOVA if significant effect was found (*p*-value ≤ 0.05 significant) |
| Okun et al.,  2009 | STN/GPi | 22 STN and 23 GPi | Prospective, double-blinded, randomized trial | Baseline and 7 months | NA | SVF and LVF | STN vs. GPi  1) STN group exhibited greater decline on letter verbal fluency (*p* = 0.03) (did not reach significance)  -Still existent in off stimulation state implying a lesion effect  Combined  1) Trend toward reduction in letter fluency (*p* = 0.07)  - Statistics performed using Hotelling's T^2^ (*p*-value ≤ 0.025 significant) |
| Ardouin et al.,  1999 | STN/GPi | 49 STN and 13 GPi | Outcomes study | Baseline and 3 months (Grenoble) or 6 months (Paris) | NA | MDRS, Grober and Buschke Test, WCST, VF, SCWT, TMT | Between sites and stimulation targets  1) Significantly higher score on the MDRS (*p* = 0.015) in STN group from Paris than STN (*p* < 0.01) and GPi (*p* < 0.01) from Grenoble  2) LVF (*p* = 0.0018) and total fluency (*p* = 0.002) decreased under stim  - observed in STN not GPi  3) TMT-A (*p* = 0.0013) and –B (*p* = 0.0015)  - Statistics performed using ANOVA, Kruskal-Wallis and Wilcoxon Tests (*p* < 0.01) |
| Pillon et al.,  2000 | STN/GPi | Group 1  48 STN and 8 GPi  Group 2  15 STN and 5 GPi | Outcomes study | Group 1  Baseline, 3 and 12 months both ON\OFF stim  Group 2  6 months (ON\OFF stim) | NA | Group 1  WCST, VF, graphic and motor series, SCWT, TMT, DRS, Grober and Buschke,  Group 2  Motor Screening, Big Little Circle, Intra and Extradimensional set-shifting, Spatial Working Memory, DOT | For STN:  1) Significant improvement on stimulation in graphic series (*p* = 0.02), SCWT (Colors: *p* = 0.02, Words: *p* = 0.0014), TMT-A (*p* = 0.04 and 0.05) and –B (notably at 12 months) (*p* = 0.01), psychomotor latency (*p* = 0.05), number of errors in spatial working memory (*p* = 0.02), and trend towards verbal working memory (*p* = 0.09)  2) Significant effect of time on CVF (poorer after surgery) (*p* = 0.0006) , TMT-B (better performance at 12 months under stimulation) (*p* = 0.01), verbal free recall (lower performance at 3 months, but recovery at 12) (*p* = 0.02)  For GPi:  1) Significant effect of time on the Initiation Subset of the DRS (improvement at 12 months) (*p* = 0.02)  STN vs GPi  1) Longer latency in simple choice reaction time for the GPi-2 group  2) Number of errors in more complex level of spatial working memory decreased under stim in STN-2 group, whereas it increased in GPi-2  3) Score on the DRS-Initiation increased at 12 months in the GPi-1, but not in STN-1 group  - Statistics performed using ANOVA for repeated measures and significant differences examined by t-tests (*p* < 0.05) |
| Trepanier et al.,  2000 | STN/GPi | 9 STN and 4 GPi bilateral | Comparison study | Baseline, 3, 6 and 12+ months | 42 unilateral pallidotomy | NART, WAIS (VIQ), PASAT, DSB, TMT-B, Spatial conditional associative learning test (executive), CVF, COWAT, CVLT, Rey-Osterrieth, Battery for Memory Efficiency (visual memory and learning), immediate recall from complex figure (visual memory and learning), serial learning of 12 designs (visual memory and learning) | 1) PASAT significantly improved in pallidotomy group and a trend was seen in the GPi-DBS group.  2) Working memory (DSB) declined in all groups, but only significant for pallidotomy  3) TMT-B, which measures set switching, declined in the whole STN-DBS group including the older group  4) Patients with left pallidotomies had increased difficulty in initial coding on CVLT, and poorer in free recall at short delay. The STN-DBS group was poorer in both free recall and cued recall at long intervals and retrieval. GPi-DBS did not have encoding difficulties but did have difficulties in long delay free and cued recall  5) PVF declined in all groups except for right pallidotomy group  6) After right pallidotomy, patients had more difficulty with complex figure, but improved to baseline levels at 6 months  7) STN-DBS group had significant reduction in the total score from six subtests on the BEM, immediate recall of complex figure, as well as learning of nonsense shapes  -Significant age effects in STN-DBS group  -Statistics performed using ANOVA (pre- and post-op for pallidotomy) and nonparametric Wilcoxon signed rank tests (DBS) |
| Rothlind et al., 2007 | STN/GPi | 23 GPi and 19 STN | Outcomes study | Baseline, 6 month unilateral | NA | COWAT, FAS, RAVLT, BVMT, WAIS (DSF, DSB, Arithmetic, Digit Symbol), TMT-A, TMT-B, SCWT, PVF, CVF, BNT, Boston Diagnostic Aphasia Examination, Complex Ideational Material | 1) Statistically significant interaction between surgical status and brain target on Digit Symbol (p=0.013)  2) After unilateral DBS for GPi and STN combined, AVF declined (*p* = 0.001), PVF declined (*p* < 0.001), and performance on Word trial of Stroop test declined (*p* = 0.018)  3) After bilateral DBS, STN had a significant decrease (*p* = 0.004) in Digit Symbol, where GPi did not  4) After bilateral, GPi group had a decline in DSB (*p* = 0.005), whereas STN did not  5) In combined bilateral DBS, reduction in AVF (*p* > 0.001), PVF (*p* = 0.021), and WAIS arithmetic (*p* = 0.018)  - Statistics performed using repeated measures ANOVA with brain target as a between-subject factor and baseline vs. follow-up as within subjects’ factor (α = 0.025) |
| Jahanshahi et al.,  2000 | STN/GPi | 7 bilateral STN/6 bilateral GPi | Outcomes study | 2-26 months post-DBS | NA | TMT, paced visual serial addition test, missing digit test, SCWT, visual conditional associative learning, WCST, random generation, word fluency | 1) No significant differences between STN and GPi groups when stimulation was off  2) Stimulation improved TMT-A (*p* = 0.003), TMT-B (*p* = 0.001), their difference (*p* = 0.006), paced visual serial addition test (*p* = 0.004), missing digit test (*p* = 0.023), control of SCWT (*p* = 0.043), and χ^2^ of random number generation (*p* = 0.005)  3) Stimulation was significantly improved by STN on TMT-B (*p* = 0.007), difference (*p* = 0.026), number of self-corrected errors (*p* < 0.001), χ^2^ of random number generation (*p* = 0.032), number of preservative errors on WCST (*p* = 0.04), non-preservative errors (*p* = 0.05)  4) Significant deterioration on conditional learning (*p* = 0.032)  - Statistics performed using repeated measures ANOVA (*p* < 0.05 significant) |

**Abbreviations**

BLOT: Benton Line Orientation Test

BNT: Bostin Naming Test

BVMT: Brief Visuospatial Memory Test

BVRT: Benton Visual Retention Test

BWR: Bi-syllabic Words Repetition Test

CBT: Corsi’s Block Tapping Test

CDT: Clock Drawing Test

CMS: Children’s Memory Scale

COWAT: Controlled Oral Word Association Test

CPT: Conners’ Continuous Performance Test

CVLT: California Verbal Learning Test

CVT: Category Verbal Fluency

DS: Digit Span

DSB: Digit Span backward

DSF: Digit Span forward

DOT: Digit Ordering Test

GNT: Graded Naming Test

HVLT: Hopkins Verbal Learning Test

HVOT: Hooper Visual Organization Test

JLOT: Judgement of Line Orientation Test

LPS: Leistungsprüfsystem

LVF: letter (lexical) fluency

(M)DRS: (Mattis) Dementia Rating Scale

MMSE: Mini-Mental State Exam

MoCA: Montreal Cognitive Assessment

(M)WCST: (Modified) Wisconsin Card Sorting Test

NART: National Art Reading Test

OMO: Odd Man Out

PAL: Paired Associate Learning

PANDA: Parkinson Neuropsychometric Dementia Assessment

PASAT: Paced Auditory Serial Addition Test

PVF: Phonemic Verbal Fluency

PWL: Paired Word Learning

(R)AVLT: (Rey) Auditory Verbal Learning Test

RCM: Raven’s Color Matrices

(R)PM-38 or 47: (Raven’s) Progressive Matrices

SCWT: Stroop Color Word Test

SDMT: Symbol Digit Modalities Test

SVF: Semantic verbal fluency

TAP: Test Battery for Assessing Attentional Disorders

TEA: Test of Everyday Attention

TMT-A: Trail Making Test-A

TMT-B: Trail Making Test-B

WAIS: Wechsler Adult Intelligence Test

WMS: Wechsler Memory Scale

WRAML: Wide Range Assessment of Memory and Learning

WRAT: Wide Range Achievement Test

VLMT: Verbal Learning Memory Test
